# Unusually Low Heat of Adsorption of CO_2_ on AlPO and SAPO Molecular Sieves

**DOI:** 10.3389/fchem.2020.588712

**Published:** 2020-10-28

**Authors:** Eduardo Pérez-Botella, Raquel Martínez-Franco, Nuria González-Camuñas, Ángel Cantín, Miguel Palomino, Manuel Moliner, Susana Valencia, Fernando Rey

**Affiliations:** ^1^Instituto de Tecnología Química, Universitat Politècnica de València-Consejo Superior de Investigaciones Científicas, Valencia, Spain; ^2^Institut Français du Pétrole (IFP) Energies Nouvelles, Lyon, France

**Keywords:** carbon dioxide, separation, adsorption, capture, molecular sieves, zeolites

## Abstract

The capture of CO_2_ from post-combustion streams or from other mixtures, such as natural gas, is an effective way of reducing CO_2_ emissions, which contribute to the greenhouse effect in the atmosphere. One of the developing technologies for this purpose is physisorption on selective solid adsorbents. The ideal adsorbents are selective toward CO_2_, have a large adsorption capacity at atmospheric pressure and are easily regenerated, resulting in high working capacity. Therefore, adsorbents combining molecular sieving properties and low heats of adsorption of CO_2_ are of clear interest as they will provide high selectivities and regenerabilities in CO_2_ separation process. Here we report that some aluminophosphate (AlPO) and silicoaluminophosphate (SAPO) materials with LTA, CHA and AFI structures present lower heats of adsorption of CO_2_ (13–25 kJ/mol) than their structurally analogous zeolites at comparable framework charges. In some cases, their heats of adsorption are even lower than those of pure silica composition (20–25 kJ/mol). This could mean a great improvement in the regeneration process compared to the most frequently used zeolitic adsorbents for this application while maintaining most of their adsorption capacity, if materials with the right stability and pore size and topology are found.

## Introduction

Carbon dioxide is a greenhouse gas that is emitted to the atmosphere due to a large number of industrial processes. Combustion of fossil fuels for transport or in power plants, metallurgy, cement and chemical production are some of the most important processes related to CO_2_ massive release to the atmosphere (U.S. National Coal Council, 2015). In order to mitigate CO_2_ emissions and prevent the negative effect they have on climate change, Carbon Capture and Storage (CCS) technologies are being applied and developed. CCS from air is the only way of reducing CO_2_ presence in the atmosphere, while CCS from large point sources (i.e., power plant or cement factory exhaust) is the best way of minimizing future emissions (Boot-Handford et al., 2014; Leung et al., 2014; The National Academies of Sciences, Engineering and Medicine, 2018).

The currently most common technology for CCS from large point sources is amine scrubbing, which involves flowing the CO_2_-containing mixture through a liquid solution of amines and its thermal regeneration afterwards. This is a highly energy demanding process, which as well presents problems with reactant stability and corrosion of the equipment. Thus, along with optimization of this technology in what refers to heat integration and reactant improvement, a number of alternative methods is being researched and developed, such as oxycombustion or separation using either membranes or adsorbents (Boot-Handford et al., 2014; Rubin et al., 2015; Bui et al., 2018; Global CCS Institute, 2019).

Separation by adsorption is being researched, as its operation costs have potential to be lower than those of other current processes. Materials that have been studied as adsorbents for CO_2_ include carbonaceous materials, metal-organic frameworks (MOFs), covalent organic frameworks (COFs), supported amines, zeolites, AlPOs, and SAPOs (Lee et al., 2008; Tagliabue et al., 2009; Liu et al., 2011; Cheung et al., 2012; Lee and Park, 2015; Fischer, 2017; Riboldi and Bolland, 2017). Selectivity, working capacity and easiness of regeneration are the three parameters to be maximized in the selection of an adsorbent. Out of the mentioned materials, supported amines and some MOFs and low silica zeolites interact chemically with CO_2_, i.e., chemisorption takes place. The CO_2_/CH_4_ and CO_2_/N_2_ selectivities on these materials is usually very high, due to the specific interaction between the CO_2_ and the adsorbent. However, this strong interaction also leads to large amounts of energy required for regeneration. These kinds of adsorbents are usually hydrophilic (or even water-sensitive) too, which is another major drawback, as water and CO_2_ adsorption interfere with each other.

Adsorption of CO_2_ on high and pure silica zeolites, together with carbonaceous materials, many MOFs and AlPOs and SAPOs takes place via a physisorption mechanism, which means that the interaction between sorbent and sorbate is weaker, thus meaning regeneration will be less energy intensive. Nevertheless, this does not mean that CO_2_/CH_4_ and/or CO_2_/N_2_ selectivities have to be low. Compositional and structural tuning of carbonaceous materials, MOFs (Lee and Park, 2015), zeolites, AlPOs, and SAPOs (García et al., 2014; Fischer, 2017) can lead to materials with high selectivities, capacities and low regeneration energies.

AlPOs and SAPOs are microporous materials closely related to zeolites and present a framework based on alternating PO_4_ and AlO_4_ tetrahedra. In the case of SAPOs, some P atoms can be replaced by isolated Si atoms and pure silica domains (Si-rich domains or Si-islands) can also be present (Man et al., 1991). These materials were discovered in the early 1980s (Wilson et al., 1982; Lok et al., 1984). Since then, AlPOs and SAPOs with many different structures have been prepared and studied as catalysts or adsorbents (Zibrowius et al., 1992; Martin et al., 1998; Schreyeck et al., 1998; Wright and Connor, 2008; Liu et al., 2011; Cheung et al., 2012; Martínez-Franco et al., 2015; Dawson et al., 2017; Fischer, 2017).

In this work, we have studied the adsorption of CO_2_ on AlPOs and SAPOs with LTA, CHA, and AFI structures (Baerlocher and McCusker), and compared the calculated isosteric heats of adsorption with those of analogous zeolites previously reported. The choice of adsorbent structures was made in order to include two small pore structures (LTA and CHA) which have been extensively reported as selective CO_2_ adsorbents (Tagliabue et al., 2009; Palomino et al., 2010; Miyamoto et al., 2012; Shang et al., 2012; Pham et al., 2014). Materials with AFI structure were included in order to check if the same findings obtained for small pore materials applied as well to large pore zeotypes. The surprisingly low isosteric heats of adsorption found in these materials suggest that AlPOs and SAPOs can present major advantages in the field of CO_2_ separation and adsorption in comparison to zeolites, if materials with structures that maximize selectivities over methane or nitrogen are found.

## Materials and Methods

### Synthesis of Materials

Experimental details on the synthesis procedures used can be found in the Supplementary Material. The naming of the samples has been explained in the SM, as well. Zeolites with LTA structure and different aluminum content were prepared as reported in IZA, Küehl (1980), Moscoso et al. (2003), Corma et al. (2004), Palomino et al. (2010), and Lemishko et al. (2016). AlPO-42, with LTA structure, was synthesized according the method reported in Schreyeck et al. (1998) and SAPO-42 materials with different Si distribution were prepared as reported in Martínez-Franco et al. (2015). Pure silica CHA and CHA-19 were synthesized using a previously reported method (Díaz-Cabañas and Barrett, 1998). Other CHA zeolitic samples containing Al (CHA-3; CHA-18) were synthesized according to procedures reported on the Verified Syntheses of Zeolitic Materials of the International Zeolite Association (VSZM-IZA) (Bourgogne et al., 1985; Zones and van Nordstrand, 1988; IZA) and CHA-6 was synthesized according to Zones (1991). SAPO-34-10 was prepared following a recently described procedure using tetraethylammonium hydroxide (TEAOH) as the OSDA (Martínez-Franco et al., 2016). SAPO-34-7 was also synthesized according to the procedure reported on the VSZM-IZA (Prakash and Unnikrishnan, 1994; IZA). Zeolite SSZ-24 (pure silica AFI) was synthesized according to a procedure reported on the VSZM-IZA (Nordstrand et al., 1988; IZA). AlPO-5 and SAPO-5 materials were synthesized following a procedure reported on the VSZM-IZA (Young and Davis, 1991; Girnus et al., 1995; IZA).

AlPO-34 was synthesized according to a novel method using (S)-1-methyl-2-(pyrrolidin-1-ylmethyl)pyrrolidine (Supplementary Figure 2) as the organic structure directing agent (OSDA). The OSDA was dispersed in a solution of phosphoric acid in water. Aluminum isopropoxide was then added and the resulting mixture stirred for 2 h at room temperature for homogenization. Hydrofluoric acid was then added, reaching pH = 7 and the resulting mixture was stirred for 1 h at room temperature. The resulting gel composition was:

$$\begin{array}{l} 1{\text{Al}}_{2}{\text{O}}_{3}:1.3\;{\text{P}}_{2}{\text{O}}_{5}:1.6\text{ OSDA}:1.3\text{ HF}:425\;{\text{H}}_{2}\text{O} \end{array}$$

The gel was introduced in a Teflon lined autoclave and kept at 175°C for 18 h with no stirring. The solid was recovered by filtration and after thorough washing with water, dried in an oven at 100°C. The resulting solid was calcined in air at 650°C.

### Characterization

Structural characterization of the studied materials was performed by powder X-Ray Diffraction (XRD) using a CUBIX PANalytical diffractometer, operating with CuKα radiation (λ_1_ = 1.5406 Å) at 45 kV and 40 mA in the 2θ range from 4 to 40°. Some samples were measured after dehydration by applying heat and under dry air flow using an *in situ* reaction chamber Anton-Paar XRK-900 attached to a PANalytical Empyrean diffractometer with CuKα radiation (λ_1_ = 1.5406 Å) at 45 kV and 40 mA in the 2θ range from 3 to 75°.

The connectivity and chemical environment of the framework species were studied by magic angle spinning nuclear magnetic resonance (MAS NMR) spectroscopy at room temperature. A Bruker Avance III HD 400 MHz spectrometer was used for this purpose. ^27^Al MAS NMR were recorded at ν_0_(^27^Al) = 104.21 MHz at a spinning rate of 20 kHz with a 90° pulse length of 1.3 μs with a 1 s repetition time. The ^27^Al chemical shift was referred to Al(NO_3_)_3_·9H_2_O. ^31^P NMR spectra were recorded at ν_0_(^31^P) = 161.9 MHz using spinning rate of 10 kHz, a 90° pulse length of 3.7 μs with spinal proton decoupling and a repetition time of 20 s. The ^31^P chemical shift was referred to phosphoric acid. ^29^Si NMR spectra were recorded at ν_0_(^29^Si) = 79.5 MHz using a spinning rate of 5 kHz with a 60° pulse length of 3.5 μs, spinal proton decoupling and 180 s as repetition time. The ^29^Si chemical shift was referred to tetramethylsilane.

The chemical composition of the solids was analyzed by inductively coupled plasma optical emission spectroscopy (ICP-OES) using a Varian 715-ES device.

Scanning electron microscopy (SEM) images were obtained using a Zeiss Ultra 55 microscope with an accelerating voltage of 1 kV.

The textural analysis was performed by measuring N_2_ isotherms at 77 K on volumetric Micromeritics ASAP 2020 and 2420 devices after activation at 400°C and under vacuum. The Brunauer-Emmet-Teller (BET) and t-plot methods were used in order to obtain estimations of the surface area and the micropore volume, respectively (Thommes et al., 2015). In some samples, the Dubinin-Astakhov (DA) method was used to calculate the surface area from CO_2_ isotherms at 273 K (Dubinin, 1975).

### Adsorption Isotherms and Calculated Isosteric Heat of Adsorption of CO_2_

CO_2_ was purchased from Abelló-Linde with 99.9993% purity. Adsorption isotherms of CO_2_ at temperatures ranging from 0 to 60°C were recorded up to 1 bar using a volumetric Micromeritics ASAP2010 and at higher pressures using a Hiden IGA3. The measured isotherms could be successfully fitted by either Virial, Toth, or Dual Site Langmuir models, which were used in the calculation of the isosteric heat of adsorption.

The procedure for the calculation of the isosteric heat of adsorption departs from the isotherms measured at 3 or more different temperatures. These isotherms are fitted to different isotherm models and the best fit is used for selecting pressure (P) values at constant loadings (Q) and different temperatures (T). Linear interpolation is also a valid way of doing this. The isosteric heat of adsorption (q_st_) at each Q is then calculated, following Clausius-Clapeyrons' equation (Equation 1).

(1) $$\begin{array}{l} q_{st}=-R{\left[\frac{\partial \ln\;P}{\partial \frac{1}{T}}\right]}_{Q} \end{array}$$

Where R is the ideal gas constant.

### Estimated Framework Negative Charge

The estimated framework negative charge is a parameter that we have defined in order to be able to compare the isosteric heats of adsorption of materials with different chemical composition and connectivities. In the case of zeolites, it equals the Al/(Si + Al) molar ratio calculated from the ICP-OES results. In the case of SAPOs, the estimated framework negative charge is calculated by combining the ICP-OES with the ^29^Si MAS NMR analyses results. The Si/(Si + Al + P) ratio is obtained from the ICP-OES data, and it gives the Si fraction out of the tetrahedral framework atoms. However, due to the different possible substitution patterns of Si in SAPOs (as single or isolated Si atoms or as Si-rich domains or SiO_2_-islands), not all the Si atoms contribute equally to the framework charge. More specifically, isolated Si atoms contribute with 1 negative charge per substituted P atom, whilst the contribution of a Si-rich domain is proportionally smaller and depends on its size. In order to differentiate between both types of Si in SAPOs, we have fitted the ^29^Si NMR spectra using two Gaussian functions centered at values between −100 and −120 ppm for SiO_2_-islands and at ca. −90 ppm, for isolated Si species. The Gaussian functions are integrated and the fraction of isolated Si is calculated. This fraction is multiplied times the Si/(Si + Al + P) ratio obtained from the ICP-OES data and the result is taken as the estimated framework negative charge. The minor contribution of the Si-islands was disregarded. The estimated framework negative charge of AlPOs and pure-silica zeolites is taken as zero.

## Results

### Characterization

XRD analysis confirmed the structural purity and degree of crystallinity of all of the calcined and/or dry materials (see Supplementary Figure 3).

Compositional analysis using ICP was used to determine the content of Al, Si, and P in all the samples (Table 1).

**Table 1:** Framework composition as determined per ICP, isolated Si fraction as determined from ^29^Si-NMR spectra and the estimated framework negative charge.

**Sample**	**x_**Si**_**	**x_**Al**_**	**x_**P**_**	**Isolated Si**	**Estimated**
				**fraction**,	**framework**
				**from NMR**	**negative charge**
AlPO-42	–	0.53	0.47	–	0
SAPO-42-104	0.04	0.50	0.46	0.24	0.010
SAPO-42-24	0.05	0.52	0.43	0.82	0.041
SAPO-42-13	0.1	0.54	0.36	0.8	0.080
Si-LTA	1	–	–	–	0
LTA-31	0.97	0.03	–	–	0.032
LTA-6	0.83	0.17	–	–	0.167
LTA-4.5	0.78	0.22	–	–	0.222
LTA-3	0.67	0.33	–	–	0.333
AlPO-34	–	0.57	0.43	–	0
SAPO-34-10	0.10	0.55	0.35	0.975	0.097
SAPO-34-7	0.18	0.51	0.31	0.814	0.146
Si-CHA	1	–	–	–	0
CHA-19	0.95	0.05	–	–	0.052
CHA-18	0.94	0.06	–	–	0.055
CHA-6	0.84	0.16	–	–	0.164
CHA-3	0.65	0.35	–	–	0.348
AlPO-5	–	0.54	0.46	–	0
SAPO-5-46	0.04	0.53	0.43	0.6	0.022
SAPO-5-34	0.07	0.54	0.40	0.45	0.030
Si-AFI	1	–	–	–	0

Solid State NMR analysis of ^27^Al, ^29^Si, and ^31^P was used in order to study the local environments of the different framework atoms. All the recorded spectra can be found in Supplementary Figures 4–6. Aluminum was found with tetrahedral coordination (50–30 ppm) but also pentacoordinated (25–10 ppm) and octahedral (0 to −20 ppm) in AlPOs and SAPOs after calcination. In addition, several signals are observed in the ^31^P spectra of AlPOs and SAPOs. These observations are primarily due to hydration of the framework, however hydration issues lay outside of the scope of this work. The ^29^Si NMR analysis allowed us to determine how much of the Si could be found as SiO_2_-islands (−105 to −120 ppm), and how much of it was isolated, i.e., surrounded by 4 Al (−80 to −100 ppm).

The estimated framework negative charge was obtained as described in section Estimated Framework Negative Charge for the different materials and the values obtained are listed on Table 1. The results are discussed along with the isosteric heats of adsorption in section Isosteric Heats of Adsorption of CO_2_.

Nitrogen adsorption at 77K was used in most samples to obtain their BET surface area applying Rouquerol's criterion and the micropore volumes using the t-plot method. However, in some zeolites with high Al content (and thus, smaller pores due to the presence of extraframework cations), nitrogen could not enter the porosity in these conditions. In those samples the surface area was calculated from the isotherm of CO_2_ at 273 K following Dubinin-Astakhov's (DA) method. In some samples, especially when the CO_2_ isotherm was close to saturation, both values were obtainable and are well-comparable. The results are presented in Table 2. It can be seen that for materials with the same structure, the calculated BET surface area and the t-plot micropore volume values are very similar. The DA surface area, however, presents larger differences between isostructural samples. In the case of LTA-4.5 (680 m^2^ g^−1^) and LTA-3 (609 m^2^ g^−1^) the reason may be the different available Na-sites for adsorption and their specific interaction with CO_2_. In the case of CHA-6 (600 m^2^ g^−1^) and CHA-3 (470 m^2^ g^−1^) the difference is remarkable and, although the interactions with some adsorption sites (Na and K ions, respectively) may be different, the presence of potassium itself reduces the average pore size thus diminishing the accessible area for CO_2_ and any other adsorbate. In fact in the CHA-3 sample, N_2_ could not enter the pores at 77 K.

**Table 2:** Textural properties of the studied adsorbents.

**Sample**	**BET surface**	**Micropore volume**	**DA surface**
	**area (m^**2**^/g)**	**(cm^**3**^/g)**	**area (m^**2**^/g)**
AlPO-42	774	0.290	–
SAPO-42-104	797	0.301	–
SAPO-42-24	776	0.289	–
SAPO-42-13	743	0.275	–
Si-LTA	811	0.320	–
LTA-31	777	0.305	–
LTA-6	806	0.297	–
LTA-4.5	799	0.304	680
LTA-3	794	0.295	609
AlPO-34	–	0.226	–
SAPO-34-10	595	0.210	–
SAPO-34-7	699	0.242	–
Si-CHA	821	0.296	–
CHA-19	869	0.305	–
CHA-18	801	0.293	–
CHA-6	749	0.273	600
CHA-3^*^	–	–	470
AlPO-5	310	0.117	–
SAPO-5-46	383	0.141	–
SAPO-5-34	355	0.119	–
Si-AFI	359	0.130	–

* *N_2_ isotherms at 77 K could not be obtained due to very slow diffusion*.

### CO_2_ Isotherms

**Figure 1:**
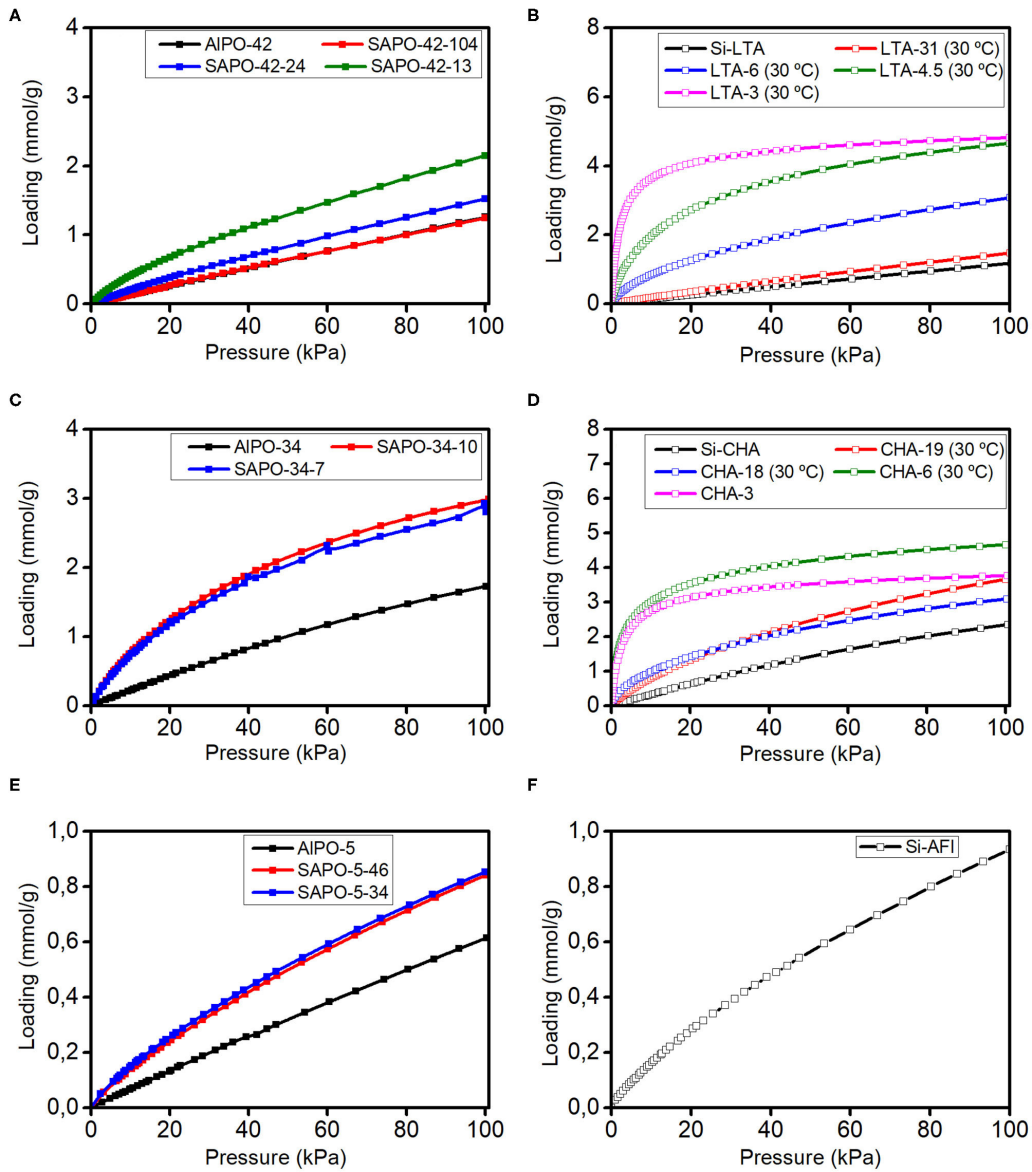
CO_2_ adsorption isotherms at 25–30°C and up to 1 bar on materials with LTA **(A,B)**, CHA **(C,D)**, and AFI **(E,F)** structures. The lines are guides to the eye.

The recorded CO_2_ isotherms at 25°C and up to 1 bar are presented in Figures 1A–F. Isotherms at different temperatures can be found in the SM. A general trend that we observe is that the higher the framework charge (proportional to the Al content in zeolites and to the amount of isolated Si in SAPOs), the steeper the low pressure regime of the isotherm. This is already an indicative of what we will find when comparing the adsorption heats. Moreover, the adsorption isotherms of AlPOs and SAPOs are similar to those of high Si/Al zeolites and reach saturation at much higher pressures (above 5 bar) than low Si/Al zeolites (above 1 bar). The maximum loading at saturation that can be estimated from the isotherms on LTA and CHA materials is comparable in all materials with the same structure and in both cases around 5 mmol/g.

### Isosteric Heats of Adsorption of CO_2_

**Figure 2:**
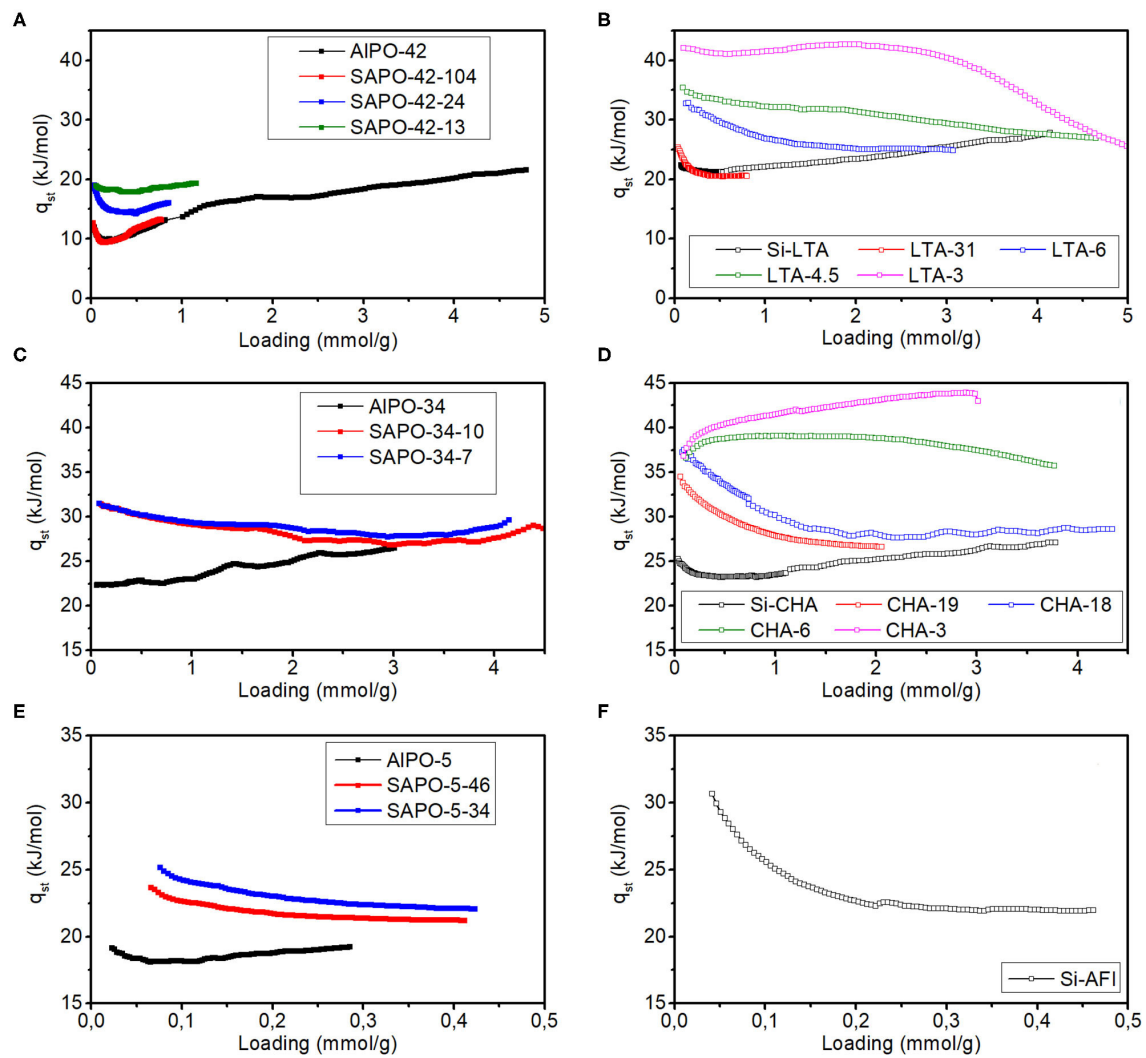
CO_2_ isosteric heat of adsorption depending on the loading on materials with LTA **(A,B)**, CHA **(C,D)**, and AFI **(E,F)** structures.

The isosteric heat of adsorption of all materials was calculated at different loading values (Q) using the linear interpolation method for the sake of comparability, as the best isotherm fit differed from case to case. The results are plotted in Figures 2A–F. An initial drop of the q_st_ with Q is seen in most AlPO and SAPO materials, and it is related to energetic inhomogeneities on the adsorbent surface. After reaching a minimum value, the q_st_ rises slowly with loading, this meaning that the lateral interactions between CO_2_ molecules are stronger than the adsorbate-adsorbent interactions (Sircar and Myers, 2003). Zeolites with high Al contents tend to present just the opposite behavior. It is noteworthy that all the q_st_ trends at high loading approach slowly values between 25 and 30 kJ/mol, which are close to the sublimation enthalpy of CO_2_. This indicates that at high loadings, the lateral interactions between CO_2_ molecules may resemble those that can be found in solid CO_2_ (U.S. Secretary of Commerce).

AlPO-42 and SAPO-42-104 present very similar isotherms and heats of adsorption (see Figure 2A), which is logical if we take into account that most of the Si in SAPO-42-104 is present as Si-islands, i.e., as “pure silica domains.” SAPO-42-13 and SAPO-42-24, both of which have predominantly isolated Si, the first having more than double the amount of Si in its framework, present a similar q_st_ starting value but for SAPO-42-24, there is a rapid decrease of the q_st_ to a value ca. 3 kJ/mol lower (see Figure 2A). The q_st_ of zeolites with this structure is in all cases well above that of SAPO and AlPO materials and follows the expected trend.

The q_st_ on AlPO-34 is a bit lower than on its pure silica counterpart at low loadings but they follow a very similar trend at loadings above 0.5 mmol/g (see Figure 2C). Materials SAPO-34-10 and SAPO-34-7 (see Figure 2C), together with zeolites CHA-19 and CHA-18 (see Figure 2D) present q_st_ trends that descend initially and are in the range 25–35 kJ/mol. Zeolites CHA-6 and CHA-3, with the highest Al contents, present q_st_ values above 35 kJ/mol, that initially increase (see Figure 2D).

All materials with AFI structure present an initial decrease of the q_st_, with the AlPO-5 having the lowest values, notably below the SAPOs and Si-AFI (see Figures 2E,F). SAPO-5-46 has a lower q_st_ than SAPO-5-34 in the whole range (see Figure 2E), as would be expected from their respective framework charges.

The mentioned general trend of SAPOs and AlPOs is interesting for their application as CO_2_ adsorbents, not only because of their generally low q_st_ values, which will result in an easy regeneration of the adsorbent, but also because of the increase of q_st_ with loading above 1 mmol/g. This means that desorbing will be progressively easier until the minimum in q_st_ is reached.

**Figure 3:**
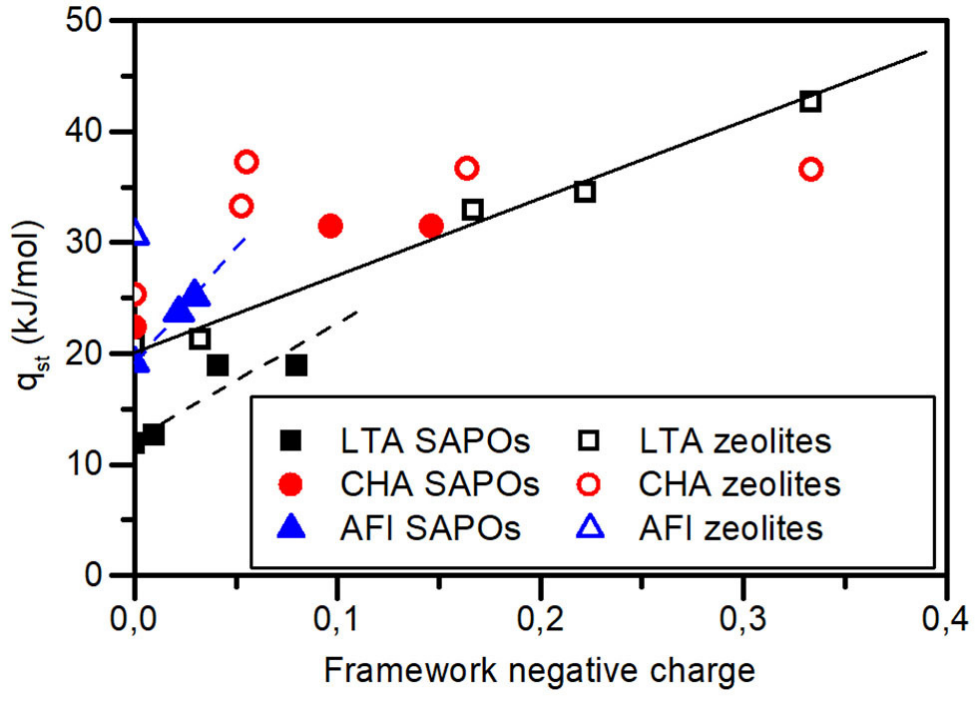
Isosteric heats of adsorption on zeolites (open symbols), AlPOs and SAPOs (filled symbols) of LTA (black squares), CHA (red circles), and AFI (blue triangles) structures at low CO_2_ loadings plotted against the estimated negative framework charge. The points labeled as SAPOs that fall in the vertical axis (zero framework charge) correspond to AlPOs.

The isosteric heats of adsorption at the lowest loadings possible have been plotted against the estimated framework negative charge (see Figure 3, Palomino's Plot), as was done previously for LTA zeolites (Palomino et al., 2010). This shows that AlPOs and SAPOs with AFI and LTA structures also present the same linear relation (discontinuous straight lines, Figure 3) seen previously in LTA zeolites (continuous straight line, Figure 3) and that these materials present notably lower heats of CO_2_ adsorption than their zeolitic counterparts, even the pure silica analogs. This is striking, as the studied pure silica zeolite samples do not present a significative amount of connectivity defects (Supplementary Figure 7) and they are usually regarded as quite inert materials. In the case of materials with CHA structure, there is also an increase of the q_st_ at low loading with increasing framework negative charge, but we cannot affirm that it is a linear relationship. On the other hand, the q_st_ of CHA zeolites seems to be higher than that of SAPOs and AlPOs with the same framework negative charge, but the trend is less clear than for the other structures.

### Comparison of AlPOs/SAPOs and Zeolites in Terms of CO_2_ and CH_4_ Adsorption

**Figure 4:**
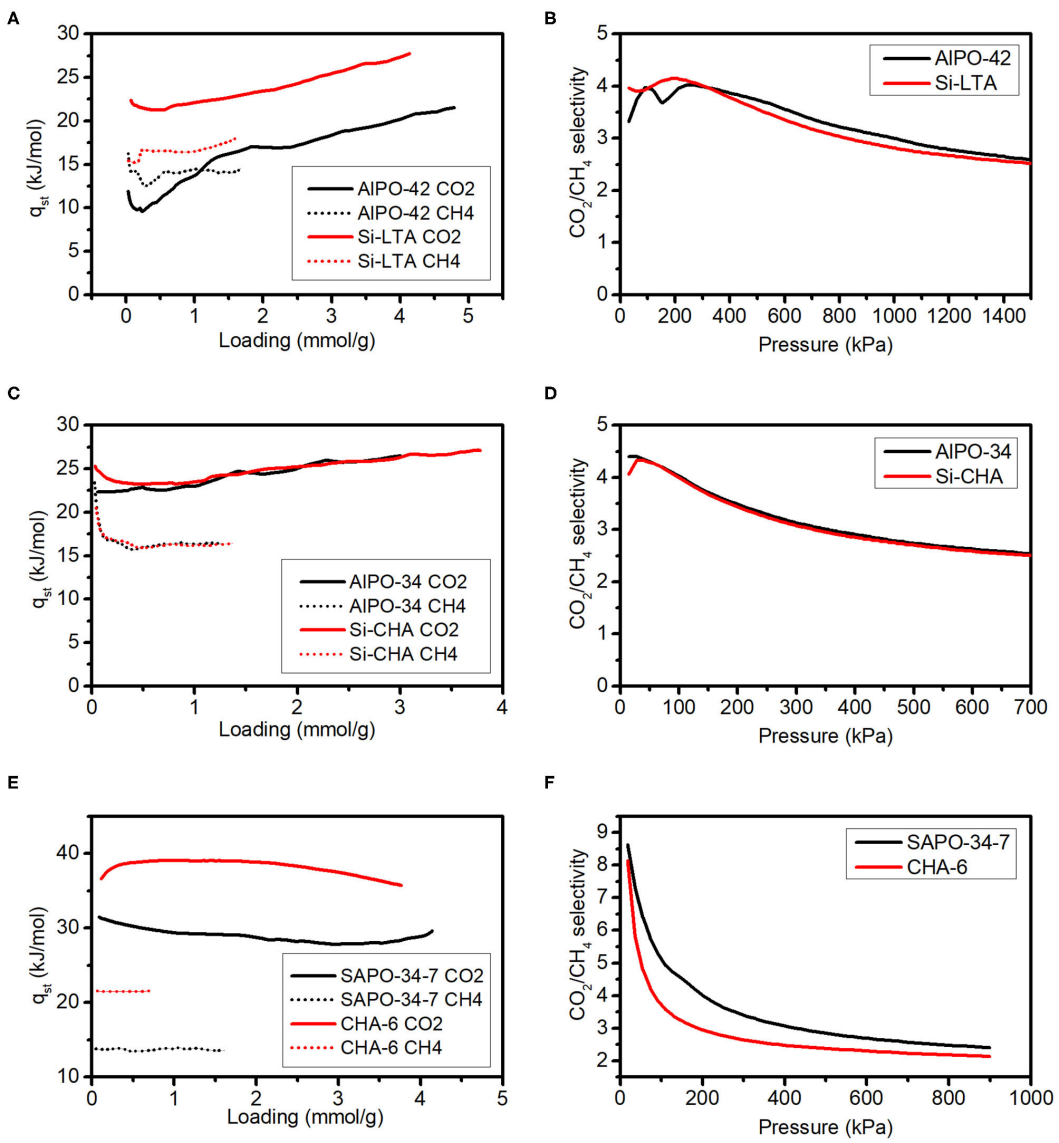
Isosteric heats of adsorption **(A,C,E)** and CO_2_/CH_4_ pure gas selectivities **(B,D,F)** on relevant AlPO/SAPO (black) and zeolite (red) pairs.

As examples of the advantages that AlPOs and SAPOs can have over zeolites in the field of CO_2_ adsorption and separation, we establish here several relevant comparisons between materials included in this study in terms of their CO_2_/CH_4_ separation ability (Figure 4 and Table 3). Materials that have been compared have the same structure and equal (or similar) estimated framework charge values, and are activated prior to adsorption at 400°C and under vacuum, which means that the differences in adsorption and selectivity will only stem from differences in the relative interactions of the adsorbates with the framework. The selectivities presented in Figure 4 have been calculated from the pure component isotherms as the ratio of adsorbed amounts at each pressure value. The CO_2_ ideal working capacities for a pressure swing adsorption (PSA) process shown in Table 3 have been calculated by subtracting the maximum loadings at 5 and 1 bar. This is an oversimplification, as in a PSA process the adsorbent will be in contact with a mixture and the adsorption of a component will be different from the pure state, but it is a useful approach to first compare different adsorbents (Bacsik et al., 2016).

**Table 3:** Carbon dioxide loadings at 1 and 5 bar of some adsorbents and their PSA working capacity.

	**Q_**1bar**_ (mmol/g)**	**Q_**5bar**_ (mmol/g)**	**Working capacity (mmol/g)**
AlPO-42	1.26	4.47	3.21
Si-LTA	1.18	4.17	2.99
AlPO-34	1.6	3.5	1.9
Si-CHA	2.24	4.62	2.38
SAPO-34-7	2.8	4.64	1.84
CHA-6	4.65	5.66	1.01

As can be seen from Figure 4A, the q_st_ of both CO_2_ and CH_4_ are lower on AlPO-42 compared to Si-LTA. The selectivity calculated as the ratio of the isotherms of the pure gases is very similar between AlPO-42 and Si-LTA (Figure 4B). Their working capacities are quite similar, with the AlPO-42 being slightly above ITQ-29. In the case of AlPO-34 and Si-CHA, the both materials are identical in terms of CH_4_ q_st_ and pure gas selectivities (Figures 4C,D), although the CO_2_ q_st_ at low loadings is lower for AlPO-34. However, the Si-CHA presents a notably higher CO_2_ adsorption capacity and also a higher working capacity. If we go to a more complex case, and compare SAPO-34-7 material with aluminosilicate zeolite CHA-6, both presenting an estimated framework charge close to 0.15, the differences in the q_st_ of both CH_4_ and CO_2_ are large and the selectivity in SAPO-34-7 is above that of CHA-6. Furthermore, going from the zeolite to the SAPO material in this case also implies a large decrease in the adsorption capacity at 1 bar, which in turn translates into a much larger working capacity of the SAPO-34-7.

With these three examples, we point out the fact that by using AlPO/SAPO adsorbents instead of zeolites, the separation of carbon dioxide from methane can be done keeping very similar maximum capacities and selectivities whilst also lowering the energy needed for regeneration.

## Conclusions

In this work we have identified a general trend according to which the isosteric heat of adsorption of CO_2_ on AlPOs and SAPOs with AFI, LTA and CHA structures is lower than on the isostructural zeolites, even of pure silica composition. This decrease in q_st_ when going from zeolites to AlPOs and SAPOs is not accompanied by a decrease in CO_2_/CH_4_ selectivity, as can be seen for materials with LTA and CHA structure. These facts lead to believe that if implemented in a CO_2_ capture process, AlPOs and SAPOs can lower the energy needed for regeneration of the adsorption bed whilst keeping the efficiency of the separation.

## Data Availability Statement

All datasets presented in this study are included in the article/Supplementary Material.

## Author Contributions

EP-B participated in the synthesis of the AFI materials and AlPO-42, performed part of the adsorption experiments, analyzed the characterization results and the adsorption data to obtain the isosteric heats of adsorption, coordinated the work of his colleagues, and wrote the main part of this work. RM-F performed the synthesis of the SAPO-42 materials and analyzed their Si-distribution. NG-C performed the synthesis of the AlPO-34 material. ÁC together with NG-C synthesized the template of the AlPO-34 material. MP performed part of the adsorption experiments and the analysis of these to obtain the isosteric heats of adsorption. MM supervised the synthesis and characterization of SAPO-42 and SAPO-34 materials, and analyzed their Si-distribution. SV supervised the synthesis and characterization of materials. FR supervised the characterization of the materials and the data analysis and coordinated the work of the whole team. All authors contributed to the article and approved the submitted version.

## Conflict of Interest

The authors declare that the research was conducted in the absence of any commercial or financial relationships that could be construed as a potential conflict of interest.
