# Supplementary material for: Unusually Low Heat of Adsorption of CO2 on AlPO and SAPO Molecular Sieves
Source: Front Chem. 2020 Oct 28;8:588712. doi: 10.3389/fchem.2020.588712 (PMC7655961; doi:10.3389/fchem.2020.588712)
Supplement: Supplementary file 1 [file Presentation_1.pdf]

frontiers

Supplementary Material

1 Nomenclature of samples

The materials have been named according to:

- **Pure silica zeolites:** "Si-" + framework type; e.g. Si-LTA is pure silica LTA zeolite
- **Aluminosilicate zeolites:** framework type + "-" + inverse of the estimated framework negative charge*; e.g. LTA-6 is an LTA aluminosilicate zeolite with estimated framework negative charge of 0.167
- **AlPOs:** "AlPO-" + number corresponding to framework type (42 = LTA, 34 = CHA, 5 = AFI); e.g. AlPO-42 is LTA AlPO
- **SAPOs:** "SAPO-" + number corresponding to framework type (42 = LTA, 34 = CHA, 5 = AFI) + inverse of the estimated framework negative charge; e.g. SAPO-42-13 is LTA SAPO with estimated framework negative charge of 0.08

*The estimated framework negative charge is a parameter that is explained in section 2.4 of the main text

2 Synthesis of materials

AlPO-42:

AlPO-42 was synthesized following a procedure similar to the one described by Schreyeck et al. (Schreyeck et al., 1998), using Kryptofix222 (K222) as the OSDA. Pseudoboehmite (Catapal A SASOL, 75 % de Al2O3), phosphoric acid (85 wt %, Sigma-Aldrich) and water were mixed and the mixture stirred for 30 min at room temperature. Hydrofluoric acid (50 wt %, Sigma-Aldrich) and K222 (99 wt %, Sigma-Aldrich) were then successively added. The gel was stirred for 14 h at room temperature. The final gel composition was:

1 Al_2O_3 : 1.03 P_2O_5 : 0.53 K222 : 0.51 HF : 81 H_2O

The resulting gel was transferred to teflon-lined stainless steel autoclaves and crystallization was carried out at 200 ºC for 1 d under static conditions. The product was then filtered off, washed with abundant deionized water and dried at 100 ºC. Calcination was carried out at 600 ºC in a tube furnace under dry air flow.

SAPO-42-13, **SAPO-42-104 and SAPO-42-24**:

These materials correspond to the LTA-4, LTA-5 and LTA-6 samples reported by Martinez-Franco et al. (Martínez-Franco et al., 2015) using 2,2-Dimethyl-2,3-dihydro-1H-benzo[de]isoquinoline-2-ium (DDBQ) and 4-methyl-2,3,6,7-tetrahydro-1H,5H-pyrido[3.2.1-ij]quinolinium (MTPQ) as the OSDAs.

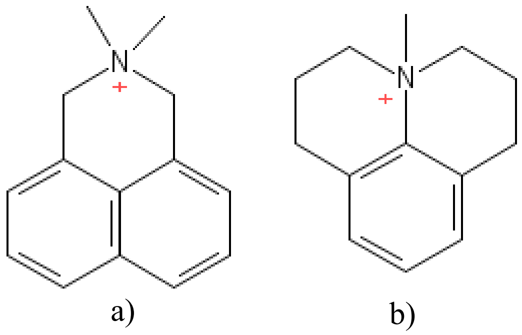

Figure S1. OSDAs used for the syntheses of SAPO-42 materials a) 2,2-Dimethyl-2,3-dihydro-1H-benzo[de]isoquinoline-2-ium (DDBQ) and b) 4-methyl-2,3,6,7-tetrahydro-1H,5H-pyrido[3.2.1-ij]quinolinium (MTPQ)

Phosphoric acid (85 wt %, Sigma-Aldrich) was added to the aqueous solution of the OSDA hydroxide. Then alumina (75 wt %, Condea) was added and the gel stirred for 5 min at room temperature. Colloidal silica (Ludox AS40, 40 wt %, Sigma-Aldrich) was added, and the mixture was stirred for 20 min. If required, hydrofluoric acid (50 wt %, Sigma-Aldrich) was finally added to the gel, and the resultant mixture was stirred for another 20 min. The gel was transferred to a teflon-lined stainless steel autoclave and heated at 175 ºC and under static conditions for 5 days. Crystalline products were filtered off, washed with abundant deionized water and dried at 100 °C. Calcination of the samples was carried out in a tube furnace using the following temperature program: the samples were heated to 580 °C using a 1 °C/min ramp under N_2 flow. The temperature was held for 8 h under dry air flow, and finally, the samples were cooled to room temperature under N_2 flow.

For the synthesis of SAPO-42-13, DDBQ was used as the OSDA, 5 wt % of previously prepared SAPO-42 crystals were added as seeds and no HF was used. The final gel composition was:

1 Al_2O_3 : 0.85 P_2O_5 : 0.30 SiO_2 : 1.85 OSDA : 111 H_2O

For the synthesis of SAPO-42-104 and SAPO-42-24, MTPQ was used as the OSDA, and the syntheses gels contained HF, with the respective gel compositions being:

1 Al_2O_3 : 0.9 P_2O_5 : 0.19 SiO_2 : 1.9 OSDA : 1.9 HF : 38 H_2O

1 Al_2O_3 : 0.9 P_2O_5 : 0.19 SiO_2 : 1.9 OSDA : 0.19 HF : 38 H_2O

Si-LTA:

Pure silica LTA zeolite, i.e. ITQ-29, was prepared following the method by Corma and coworkers (Corma et al., 2004). The gel was prepared by hydrolyzing tetraethylorthosilicate (TEOS, from Merck) in an aqueous solution of 4-methyl-2,3,6,7-tetrahydro-1H,5H-pyrido[3.2.1-ij]quinolinium (ROH) and tetramethylammonium hydroxides (TMAOH, 25 wt % aqueous solution from Sigma-Aldrich). The mixture was kept under stirring until the ethanol formed upon hydrolysis of TEOS and the appropriate excess of water were evaporated to reach the gel composition given below. Finally, an aqueous solution of HF (50 wt %, Sigma-Aldrich) was added and the mixture was introduced in a Teflon-lined stainless autoclave and heated at 135 ºC for 7 days under rotation at 60 rpm.

1 SiO_2 : 0.25 ROH : 0.25 TMAOH : 0.5 HF : 3 H_2O

After this time the mixture was filtered, washed with water, dried at 100 °C and finally calcined in air at 700 ºC.

LTA-31:

The LTA-31 zeolite was synthesized in fluoride medium following a similar method (same OSDAs) as the one described above (Corma et al., 2004; Lemishko et al., 2016). The gel was prepared by hydrolyzing TEOS (Merck) in an aqueous solution of ROH and TMAOH. Then the appropriate amount of aluminum isopropoxide (AIP, from Sigma-Aldrich) was added and the mixture was kept under stirring until ethanol and 2- propanol and the appropriate excess of water were evaporated to reach the gel composition given above. After that, an aqueous solution of HF (50 wt %, Sigma-Aldrich) was added and, finally, pure silica ITQ-29 seeds were incorporated as a suspension in water to reach 15% of the total silica. The mixture was introduced in a Teflon-lined stainless autoclave and heated at 125 °C for 3 days under rotation at 60 rpm.

1 SiO_2 : 0.01 Al_2O3 : 0.25 ROH : 0.25 TMAOH : 0.5 HF : 3 H_2O

After this time the mixture was filtered, washed with water, dried at 100 °C and finally calcined in air at 700 ºC. The calcined sample was exchanged with sodium. ICP chemical analysis revealed a Si/Al ratio of 30.

LTA-6:

The synthesis of LTA-6 was done basing on the method appearing on Example 1 in (Moscoso et al., 2003). Aluminium secbutoxide (97 wt %, Sigma-Aldrich) was added to an aqueous solution of tetraethylammonium (TEAOH, 35 wt % aqueous solution, Sigma-Aldrich) and diethyldimethylammonium (DEDMAOH, 20 wt % aqueous solution, Sigma-Aldrich) hydroxides under stirring. Colloidal silica (Ludox AS40, 40 wt %, Sigma-Aldrich) was then added and the mixture

stirred for 1 h and aged at 95 ºC overnight. An aqueous solution of TMACl (98 wt %, Sigma-Aldrich) and NaCl (Sigma-Aldrich) was then added and the mixture homogeneized for 30 min. The resulting gel was introduced in teflon-lined stainless steel autoclaves and crystallization was carried out at 100 ºC for 13 days under static conditions. The gel composition was:

1 SiO_2 : 0.05 Al_2O_3 : 0.3 TEAOH : 0.2 DEDMAOH : 0.05 TMACl : 0.05 NaCl : 17 H_2O

After this time the mixture was filtered, washed with water, dried at 100 °C and finally calcined in air at 500 ºC. The calcined sample was exchanged with sodium. ICP chemical analysis revealed a Si/Al ratio of 5.

LTA-4.5:

The synthesis of LTA-4.5 was done basing on the method appearing on Example 10 in (Moscoso et al., 2003). Aluminium sec-butoxide (97 wt %, Sigma-Aldrich) was added to an aqueous solution of DEDMAOH (20 wt %, Sigma-Aldrich) under stirring. Colloidal silica (Ludox AS40, 40 wt %, Sigma-Aldrich) was then added and the mixture stirred for 20 h and aged at 95 ºC overnight. An aqueous solution of TMACl (98 wt %, Sigma-Aldrich) and NaCl (Sigma-Aldrich) was then added and the mixture homogeneized for 30 min. The resulting gel was introduced in teflon-lined stainless steel autoclaves and crystallization was carried out at 125 ºC for 10 days under static conditions. The gel composition was:

1 SiO_2 : 0.06 Al_2O_3 : 0.85 DEDMAOH : 0.12 TMACl : 0.05 NaCl : 30 H_2O

After this time the mixture was filtered, washed with water, dried at 100 °C and finally calcined in air at 500 ºC. The calcined sample was exchanged with sodium. ICP chemical analysis revealed a Si/Al ratio of 3.5.

LTA-3:

LTA zeolite with Si/Al=2 was synthesized following a modified recipe based on that reported for zeolite Alpha in the Verified Syntheses of Zeolitic Materials of the IZA Synthesis Commission(IZA; Küehl, 1980). Sodium aluminate (54% Al_2O_3, 39% Na_2O, Carlo Erba), tetramethylammonium hydroxide (TMAOH, 25 wt % aqueous solution, Sigma-Aldrich), colloidal silica (Ludox AS40, 40 wt %, Sigma-Aldrich) and water were used for the preparation of a gel of the following molar composition:

1 SiO_2 : 0.17 Al_2O_3 : 0.78 TMAOH : 0.2 Na_2O : 33 H_2O

The synthesis mixture was aged at 35-40ºC during 24 hours prior to the crystallization at 100ºC for 28 hours. The zeolite was recovered by filtration and extensively washing with deionized water, followed by drying at 100ºC.

The zeolite was then calcined at 500ºC in static air for 3 hours to remove the occluded organic material. The calcined sample was exchanged with sodium. ICP chemical analysis revealed a Si/Al ratio of 1.9.

AlPO-34:

AlPO-34 was synthesized according to a novel method using (S)-1-methyl-2-(pyrrolidin-1-ylmethyl)pyrrolidine (figure S2) as the organic structure directing agent (OSDA).

The OSDA was prepared departing from from N-(tert-Butoxycarbonyl)-L-proline (BOC-(L)-Pro-OH). A solution of N,N'-dicyclohexylcarbodiimide (DCC) (30.5 g, 148.6 mmol) in CH_2Cl_2 (60 mL) was added to a suspension of BOC-(L)-Pro-OH (32.0 g, 148.6 mmol) in CH_2Cl_2 (45 mL) at 0º C under N_2 atmosphere and the reaction mixture was stirred for 30 minutes at that temperature. A solution of pyrrolidine (12.4 mL, 148.6 mmol) in CH_2Cl_2 (65 mL) was added dropwise to the previous reaction mixture at 0º C, and after 15 minutes the temperature was increased to room temperature. After 16 h, the mixture was concentrated under reduced pressure and ethyl acetate was added to the residue. The resulting insoluble materials were removed by filtration and the filtrate was washed with 1N HCl, saturated $NaHCO_3$ and brine successively, before drying over $MgSO_4$. Concentration of the organic phase provided the amide (35.6 g) which was used in the next step without further purification.

$LiAlH_4$ (12.6 g, 332.2 mmol) was suspended in anhydrous THF (150 mL) under N_2 atmosphere. The amide (35.6 g, 132.9 mmol) was solved in anhydrous THF (150 mL) and added dropwise at 0º C into the previous suspension through an addition funnel with pressure-equalization arm. After 30 minutes at room temperature, the mixture was heated under reflux for 20 hours. Finally, the reaction was cooled with an ice bath and quenched by addition of H_2O (10 mL), 15 wt% aqueous solution of NaOH (6 mL) and H_2O (6 mL) again. After 30 min. of stirring at room temperature the mixture was filtered and the solvent evaporated. The resulting residue was purified by chromatography using a column filled with Al_2O_3 as the stationary phase and a mixture of hexane/AcOEt 6:1 as the mobile phase. The resulting solution which contained the diamine was rotavaporated and the desired product, (S)-1-methyl-2-(pyrrolidin-1-ylmethyl)pyrrolidine was obtained with 46 % yield (10.2 g).

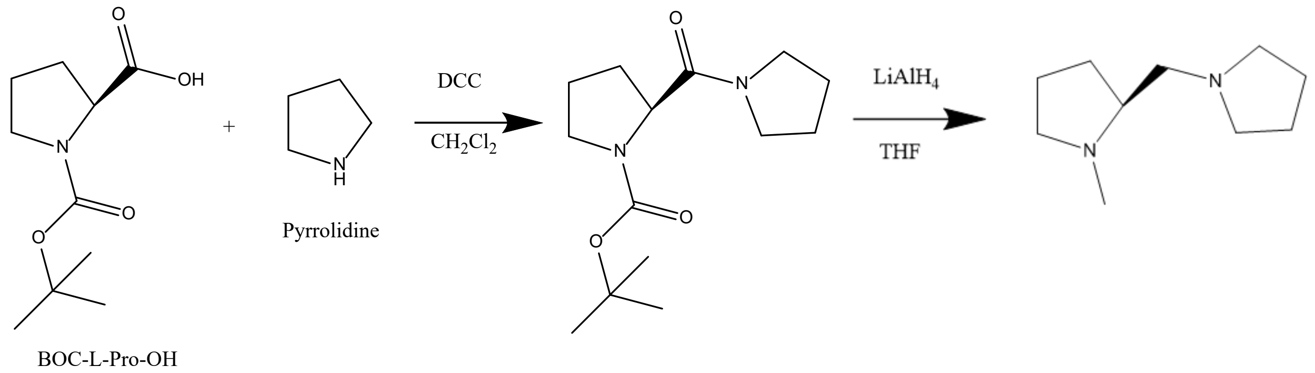

Figure S2. Scheme of the preparation of the OSDA used in the synthesis of AlPO-34 material, (S)-1-methyl-2-(pyrrolidin-1-ylmethyl)pyrrolidine

The OSDA was dispersed in a solution of phosphoric acid (85 wt %, Sigma-Aldrich) in water. Aluminium isopropoxide (99 wt %, Sigma-Aldrich) was then added and the resulting mixture stirred for 2 h at room temperature for homogenization. Hydrofluoric acid (50 wt %, Sigma-Aldrich) was then added, reaching pH = 7 and the resulting mixture was stirred for 1 h at room temperature. The resulting gel composition was:

1 Al_2O_3 : 1.3 P_2O_5 : 1.6 OSDA : 1.3 HF : 425 H_2O

The gel was introduced in a teflon lined autoclave and kept at 175 ºC for 18 h with no stirring. The solid was recovered by filtration and after thorough washing with water, dried in an oven at 100 ºC. The resulting solid was calcined in air at 650 ºC.

SAPO-34-10:

SAPO-34-10 was prepared by a previously reported method using tetraethylammonium hydroxide (TEAOH) as the OSDA (Martínez-Franco et al., 2016). Colloidal silica (Ludox AS40, 40 wt %, Sigma-Aldrich) was used as the silica source and partially hydrated alumina (75 wt %, Condea) as the aluminium source. Phosphoric acid (85 wt %, Sigma-Aldrich) was dissolved in water and TEAOH (35 wt %, Sigma-Aldrich) was added. The alumina was then added and the mixture stirred for 5 min. Finally the silica source was added and the mixture was stirred at room temperature until complete homogeneization (ca. 20 min). The resulting gel composition was:

1 Al_2O_3 : 0.8 P_2O_5 : 0.27 SiO_2 : 1.8 OSDA : 36 H_2O

The crystallization was carried out at 175 ºC for 2 days. The solids were filtered off and washed until neutral pH was reached in the washing waters. The solid was dried at 100 ºC overnight and finally calcined at 550 ºC in air.

SAPO-34-7:

The synthesis of the silicoaluminophosphate SAPO-34-7 was carried out following a procedure reported on the Verified Syntheses of Zeolitic Materials of the International Zeolite Association (IZA; Prakash and Unnikrishnan, 1994). For this preparation, alumina (Condea Pural, 74.6 wt % Al_2O_3) was slowly added to an aqueous solution of phosphoric acid and stirred during four hours. Then, a mixture containing fumed silica (Aerosil 200, Degussa), morpholine (M, 99 wt %, Sigma-Aldrich) and water was prepared and added dropwise to the former one. The overall gel was homogenized by stirring for three hours. The molar composition was the following:

Al_2O_3 : 1.06 P_2O_5 : 1.08 SiO_2 : 2.09 M : 66 H_2O

The gel was aged at 38ºC during 24 hours and later introduced in Teflon-lined stainless steel autoclaves and heated at 200ºC for 24 hours in static conditions. The autoclaves were cooled down and the sample was recovered by filtration, washing with deionized water and drying at 100ºC. It was submitted to calcination in air atmosphere at 580ºC for three hours in order to remove the occluded organic.

Si-CHA:

The synthesis of purely siliceous Chabazite was carried out in fluoride medium following a reported procedure (Díaz-Cabañas and Barrett, 1998). In particular, tetraethylorthosilicate (TEOS, 98 wt %, Merck) was added to an aqueous solution of N,N,N, trimethyl-1-adamantammonium hydroxide (ROH). The mixture was stirred during the required time in order to allow the evaporation of the ethanol formed during the hydrolysis of TEOS and the excess of water. Finally, an aqueous solution of HF (50 wt %, Sigma-Aldrich) was added and the mixture was homogenized by hand. The molar composition of the gel was the following:

SiO_2 : 0.50 ROH : 0.50 HF : 3 H_2O

The gel was introduced in Teflon-lined stainless steel autoclaves and heated at 150ºC during two days in rotating conditions. The autoclaves were cooled down and the zeolite was recovered by filtration, washing with deionized water and drying at 100ºC. The zeolite was submitted to calcination in air atmosphere at 580ºC for three hours in order to remove the occluded organic.

CHA-19:

The synthesis of Chabazite with Si/Al ratio around 15 in fluoride medium was carried out following a procedure based in that reported for the pure silica zeolite (Díaz-Cabañas and Barrett, 1998). In particular, tetraethylorthosilicate (TEOS, 98 wt %, Merck) and aluminium isopropoxide (99 wt %, Sigma-Aldrich) were added to an aqueous solution of N,N,N, trimethyl-1-adamantammonium hydroxide (ROH). The mixture was stirred during the time required for allowing the evaporation of the ethanol formed during the hydrolysis of TEOS and the excess of water and, finally, an aqueous solution of HF (50 wt %, Sigma-Aldrich) was added. The mixture was homogenized by hand and the molar composition of the gel was the following:

SiO_2 : 0.005 Al_2O_3 : 0.50 ROH : 0.50 HF : 3 H_2O

The gel was introduced in Teflon-lined stainless steel autoclaves and heated at 150ºC during four days in rotating conditions. The autoclaves were cooled down and the zeolite was recovered by filtration, washing with deionized water and drying at 100ºC. The zeolite was submitted to calcination in air atmosphere at 580ºC for three hours in order to remove the occluded organic. ICP chemical analysis revealed a Si/Al ratio of 18.

CHA-18:

The synthesis of Chabazite with Si/Al ratio around 15 was carried out following a procedure reported on the Verified Syntheses of Zeolitic Materials of the International Zeolite Association for preparing zeolite SSZ-13 (IZA; Zones and Van Nordstrand, 1988). For this preparation, an aqueous solution containing NaOH (Sigma-Aldrich) and N,N,N, trimethyl-1-adamantammonium hydroxide (ROH) was prepared. Then, aluminium hydroxide (57 wt % Al_2O_3, Sigma-Aldrich) followed by fumed silica (Aerosil 200, Degussa) were also incorporated and the mixture was homogenized by stirring in order to form a gel of the following molar composition:

SiO_2 : 0.025 Al_2O_3 : 0.20 ROH : 0.10 Na_2O : 44 H_2O

The gel was introduced in Teflon-lined stainless steel autoclaves and heated at 160ºC during four days in rotating conditions. The autoclaves were cooled down and the zeolite was recovered by filtration, washing with deionized water and drying at 100ºC. The zeolite was submitted to calcination in air atmosphere at 580ºC for three hours in order to remove the occluded organic. ICP chemical analysis revealed a Si/Al ratio of 17.

CHA-6:

Chabazite with Si/Al ratio of 5 was prepared according to (Zones, 1991). First, N,N,N, trimethyl-1-adamantammonium iodide (RI) was prepared by alkylation of 1-adamantamine (97 wt %, Sigma-Aldrich) with methyl iodide (99 wt %, Sigma-Aldrich). The required amount of RI was added to an aqueous solution of NaOH together with sodium silicate solution (26.7 wt % SiO_2, 7.9 wt % Na_2O, Supelco) and, finally, zeolite Y (CBV500, Zeolyst) was incorporated to the mixture. After homogenization of the ingredients by stirring for two hours, a gel of the following molar composition was obtained:

SiO_2 : 0.027 Al_2O_3 : 0.09 RI : 0.34 Na_2O : 25 H_2O

The mixture was introduced in Teflon-lined stainless steel autoclaves and heated at 135ºC during four days in static conditions. The autoclaves were cooled down and the zeolite was recovered by filtration, washing with deionized water and drying at 100ºC. The zeolite was submitted to calcination in air atmosphere at 580ºC for three hours in order to remove the occluded organic. ICP chemical analysis revealed a Si/Al ratio of 5.

CHA-3:

The synthesis of Chabazite with Si/Al ratio of 2 was carried out following a procedure reported on the Verified Syntheses of Zeolitic Materials of the International Zeolite Association (IZA; Bourgogne et al., 1985). In particular, commercial zeolite Y (CBV500, Zeolyst) was added to an aqueous solution of KOH in a polypropylene bottle in order to form a gel of the following molar composition:

SiO_2 : 0.19 Al_2O_3 : 0.03 Na_2O : 0.39 K_2O : 43 H_2O

The mixture was stirred for 10 minutes at room temperature and then heated at 100ºC for five days in static conditions. The zeolite was then recovered by filtration, washing with deionized water and drying at 100ºC. ICP chemical analysis revealed a Si/Al ratio of 2.

AlPO-5:

AlPO-5 was synthesized following a procedure reported on the Verified Syntheses of Zeolitic Materials of the International Zeolite Association (IZA; Girnus et al., 1995) using triethylamine (TEA) as the OSDA. Phosphoric acid (85 wt %, Sigma-Aldrich) is dissolved in water under stirring. TEA (99 wt %, Sigma-Aldrich) is added dropwise under continuous stirring. Aluminium isopropoxide (99 wt %, Sigma-Aldrich) is then slowly added while cooling at 0 ºC and stirring. The mixture is stirred at room temperature for another 2 h, or until complete evaporation of the isopropanol. Finally, aqueous HF (50 wt %, Sigma-Aldrich) is added and the mixture is stirred for further 2 h. The final gel composition was:

1 Al_2O_3 : 1.05 P_2O_5 : 2.00 TEA : 51.2 H_2O

The resulting gel was transferred to teflon-lined stainless steel autoclaves and crystallization was carried out at 200 ºC for 3 to 24 h under static conditions. The product was then filtered off, washed with abundant deionized water and dried at 100 ºC. Calcination was carried out at 600 ºC in air.

SAPO-5-46 and SAPO-5-34:

These materials were synthesized following a procedure reported on the Verified Syntheses of Zeolitic Materials of the International Zeolite Association (IZA; Young and Davis, 1991) using cyclohexylamine as the OSDA and varying the Si content of the gel. Aluminium isopropoxide (99 wt %, Sigma-Aldrich) is suspended in water to form a slurry. An aqueous solution of phosphoric acid (85 wt %, Sigma-Aldrich) was then added dropwise to the slurry under stirring. The resulting mixture is further stirred for 1 h to ensure homogeneity. Cyclohexylamine (99.9%, Sigma-Aldrich) is then added dropwise under stirring. The resulting mixture is further stirred for 1.5 h to ensure homogeneity. Finally, colloidal silica (Ludox AS40, 40 wt %, Sigma-Aldrich) is added and the mixture is stirred for another 10 min.

The final gel compositions of SAPO-5-46 and SAPO-5-34 were, respectively:

1 Al_2O_3 : 1 P_2O_5 : 0,4 SiO_2 : 2 TEA : 50 H_2O

1 Al_2O_3 : 1 P_2O_5 : 0,8 SiO_2 : 2 TEA : 50 H_2O

The resulting gel was transferred to teflon-lined stainless steel autoclaves and crystallization was carried out at 200 ºC for 3 h under static conditions. The product was then filtered off, washed with abundant deionized water and dried at 100 ºC. Calcination was carried out at 600 ºC in air.

Si-AFI:

Zeolite SSZ-24 (pure silica AFI) was synthesized according to a procedure reported on the Verified Syntheses of Zeolitic Materials of the International Zeolite Association (IZA; Nordstrand et al., 1988). A solution of trimethyl-1-adamantammonium hydroxide (6.96 % wt, 0.329 mmol OH/ g) was mixed with water and potassium hydroxide (Sigma-Aldrich) until homogeneity was reached. Fumed silica (Sigma-Aldrich) was added and the resulting gel stirred manually. The resulting gel had the composition:

1 SiO_2 : 0.06 K_2O : 0.15 ADE : 40 H_2O

The gel was transferred to teflon-lined stainless steel autoclaves and crystallization was carried out at 150 ºC for 10 days in static conditions. The crystalline solids were recovered by filtration and washed thoroughly with water until neutral pH was reached. The material was dried at 100 ºC and finally, calcined at 580 ºC in air.

3 Characterization of materials

The X-Ray diffractograms of all the calcined AlPOs, SAPOs and zeolites (except LTA zeolites) are presented in Figure S3. The NMR spectra of all the studied AlPOs and SAPOs can be found in figures S4-S6. The SEM images of selected materials can be found in Figures S7-S8. Even though the crystal sizes of the different materials vary between ca. 0.5 and 8 μm, no diffusion limitations were found.

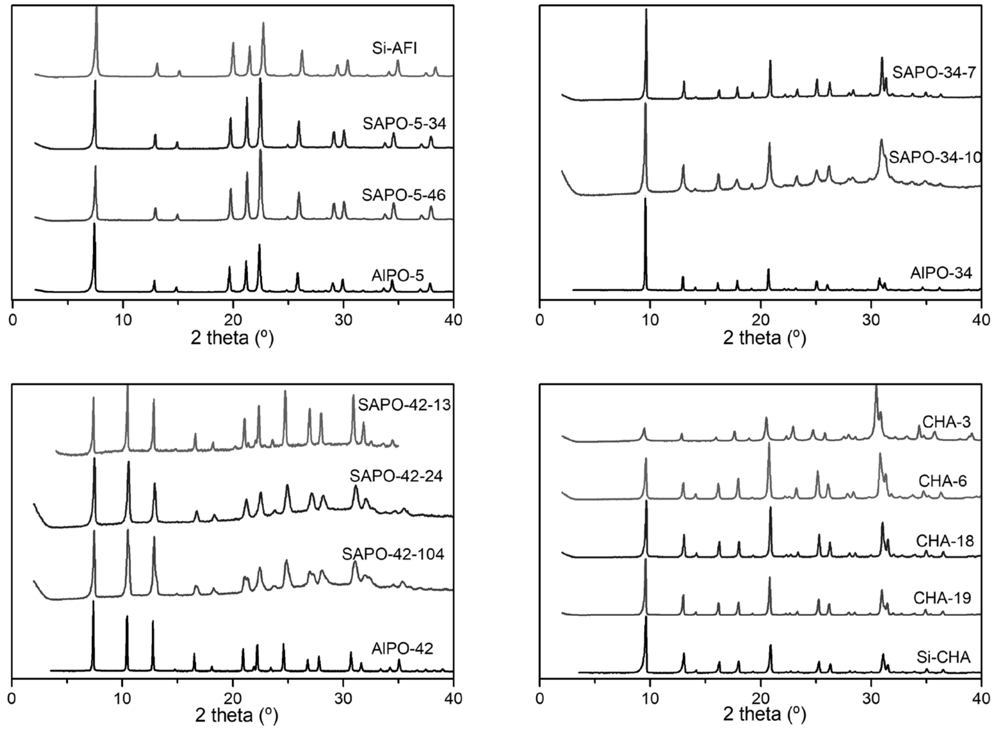

Figure S3. X-Ray diffractograms of the calcined materials studied in this work. The ones corresponding to LTA-zeolites can be found in a previous paper by Palomino et al. (Palomino 2010)

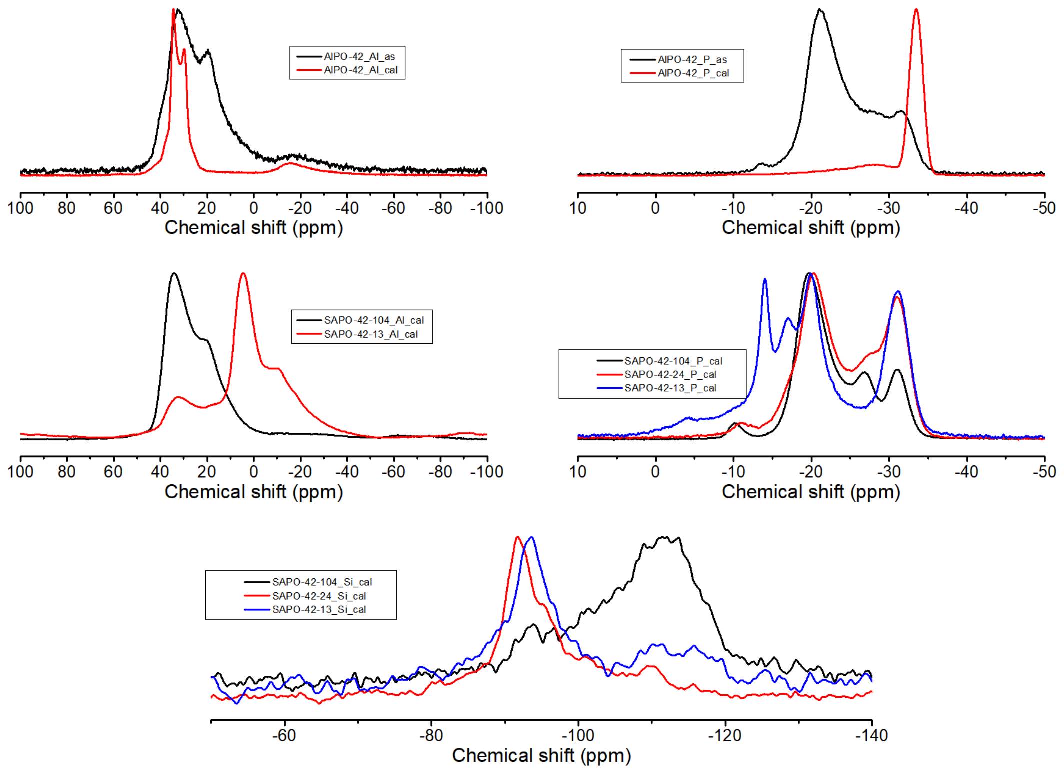

Figure S4. NMR spectra of the SAPO-42 and AlPO-42 materials studied in this work.

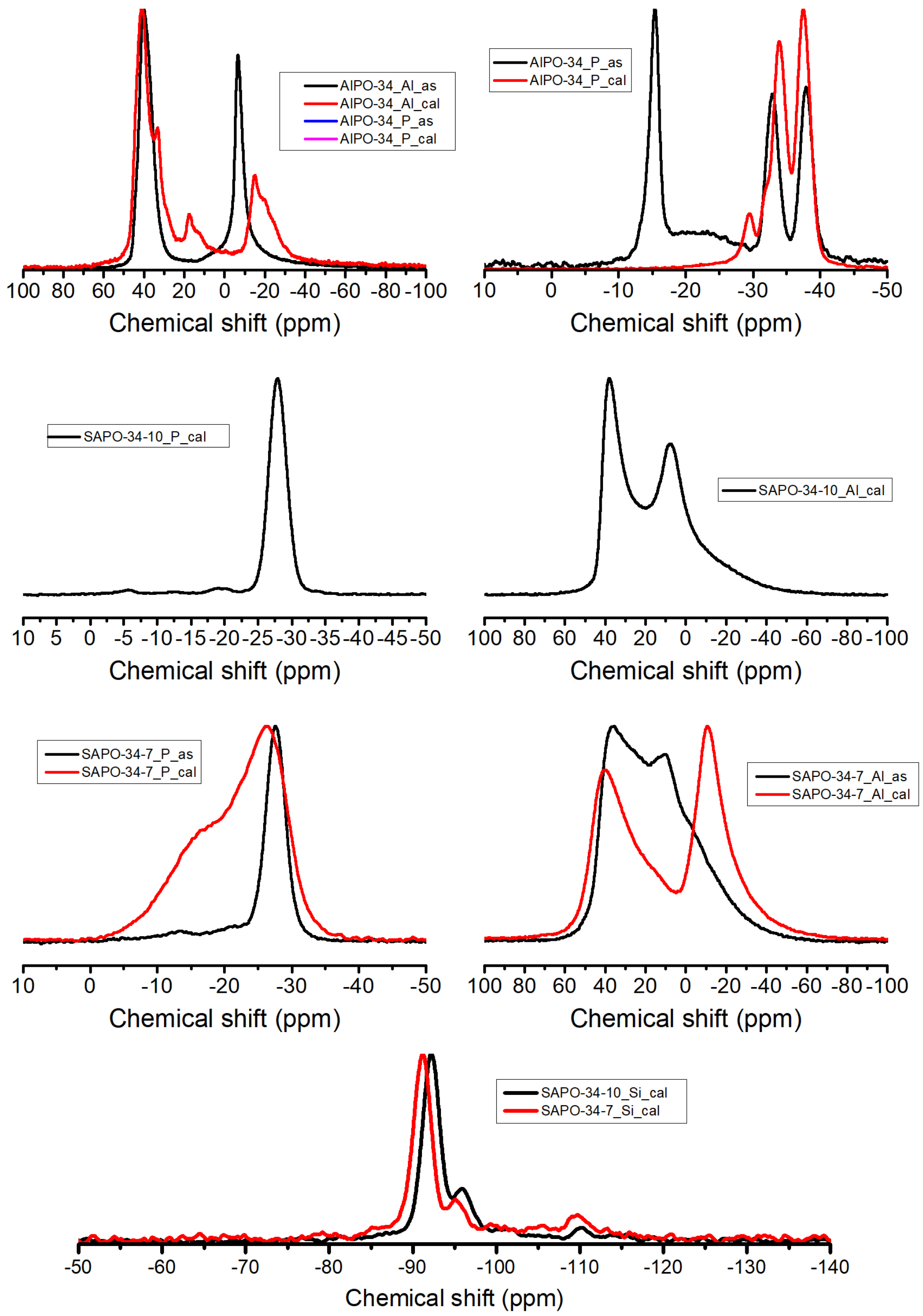

Figure S5. NMR spectra of the SAPO-34 and AlPO-34 materials studied in this work.

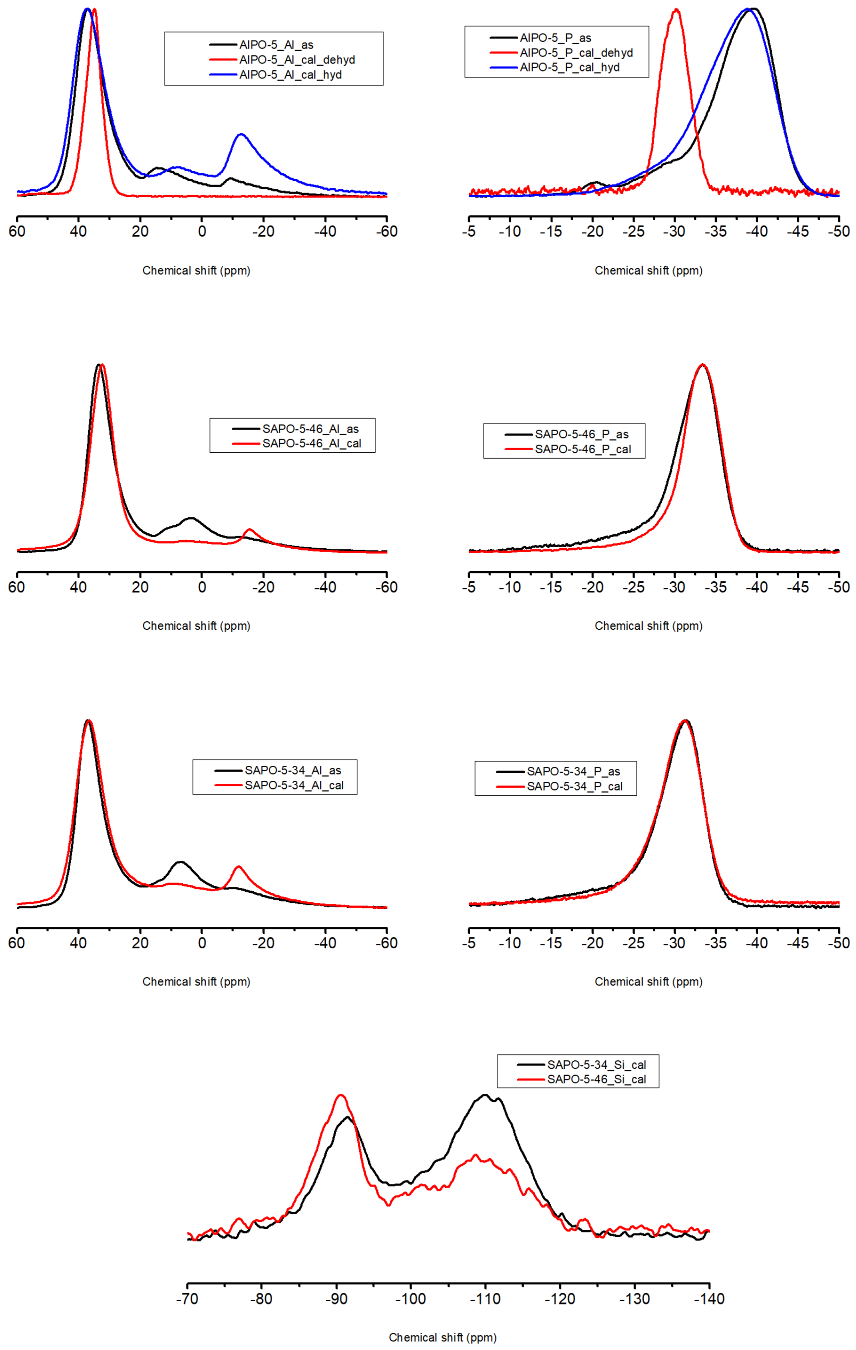

Figure S6. NMR spectra of the SAPO-5 and AlPO-5 materials studied in this work.

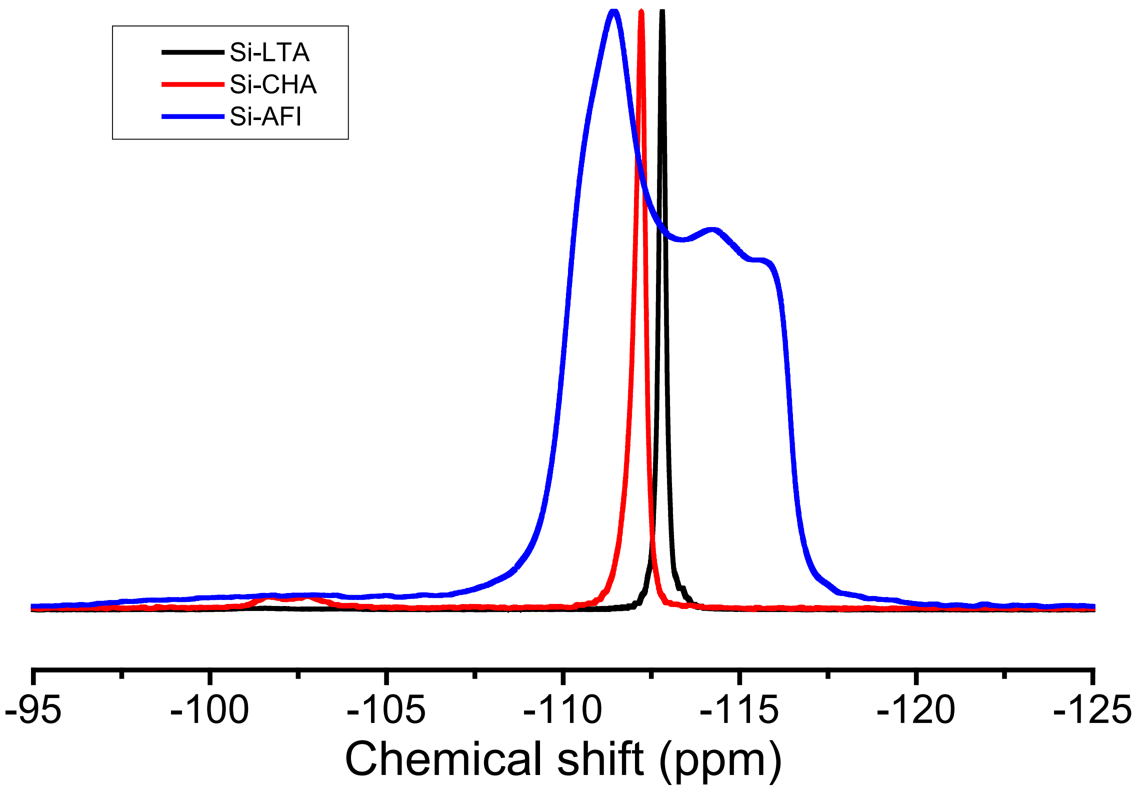

Figure S7. ^{29}Si-NMR spectra of the pure silica zeolites with LTA, CHA and AFI structures.

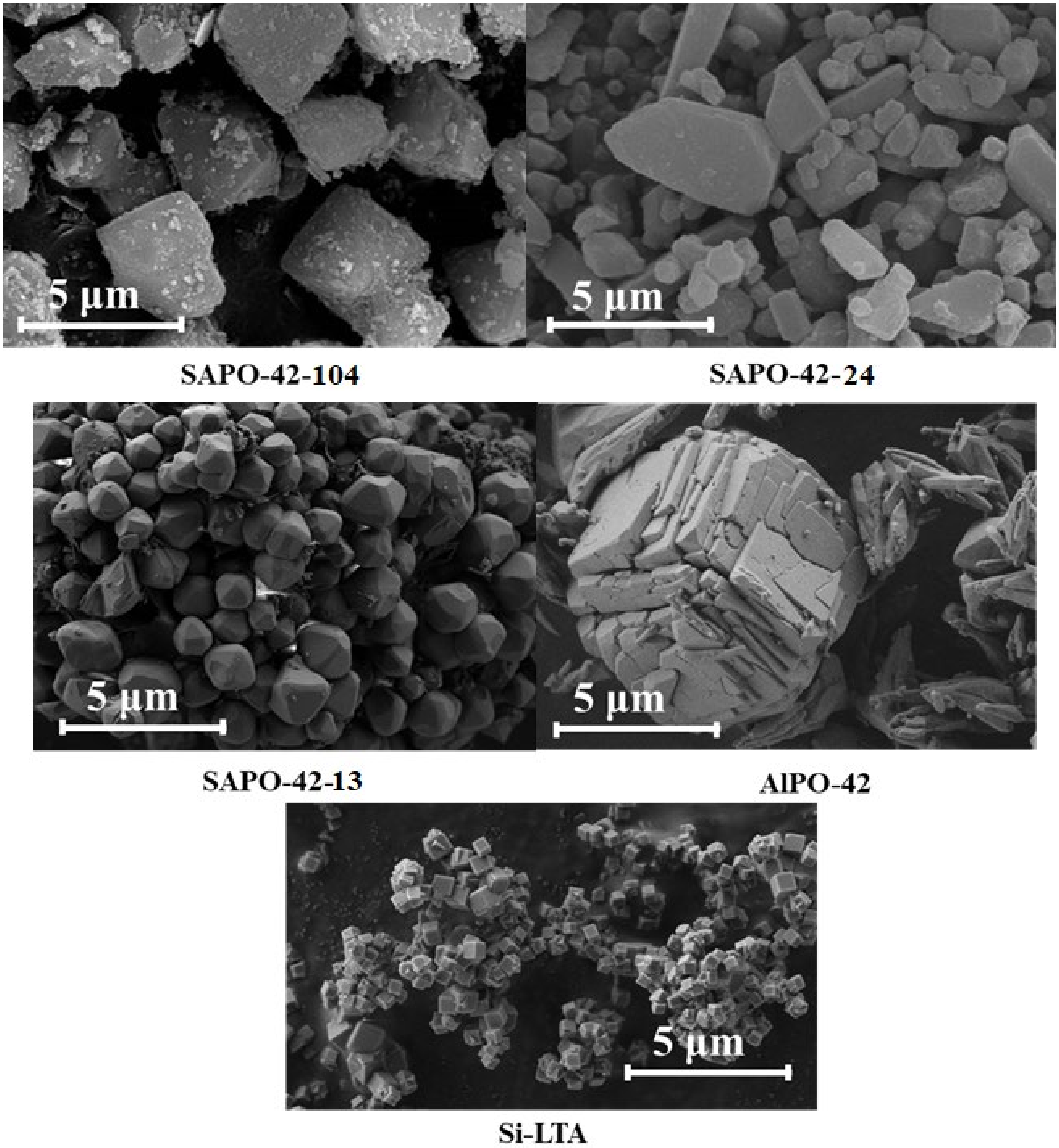

Figure S8. SEM images of materials with LTA structure.

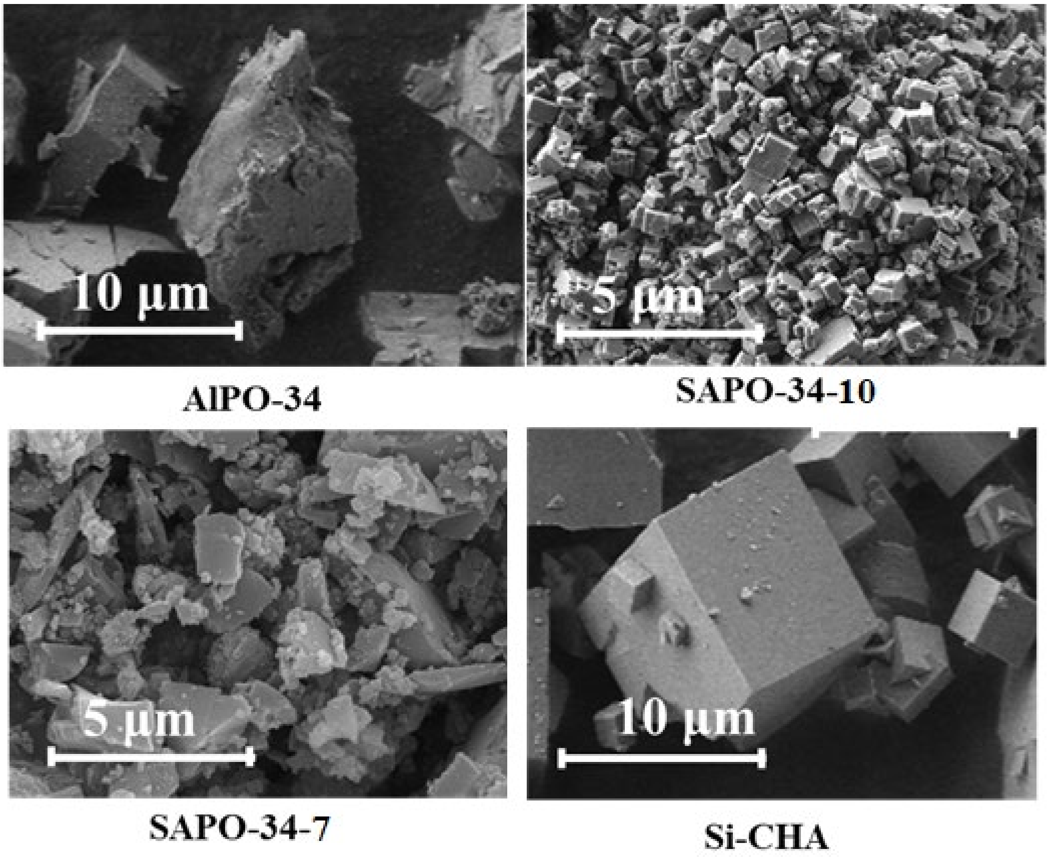

Figure S9. SEM images of materials with CHA structure.

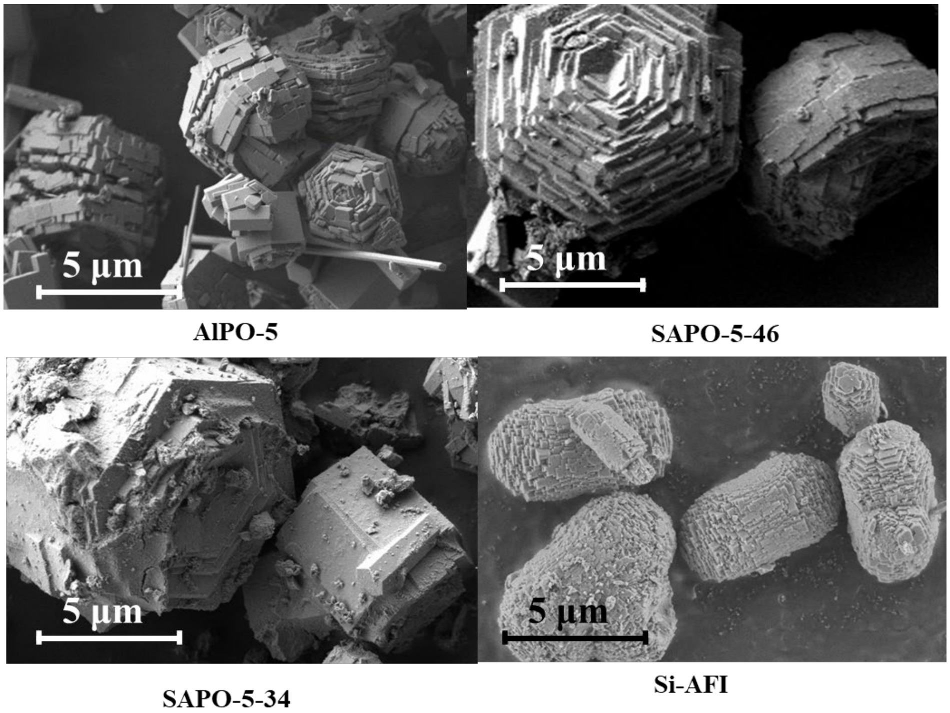

Figure S10. SEM images of materials with AFI structure.

Adsorption experiments

3.1 CO_2 Isotherms

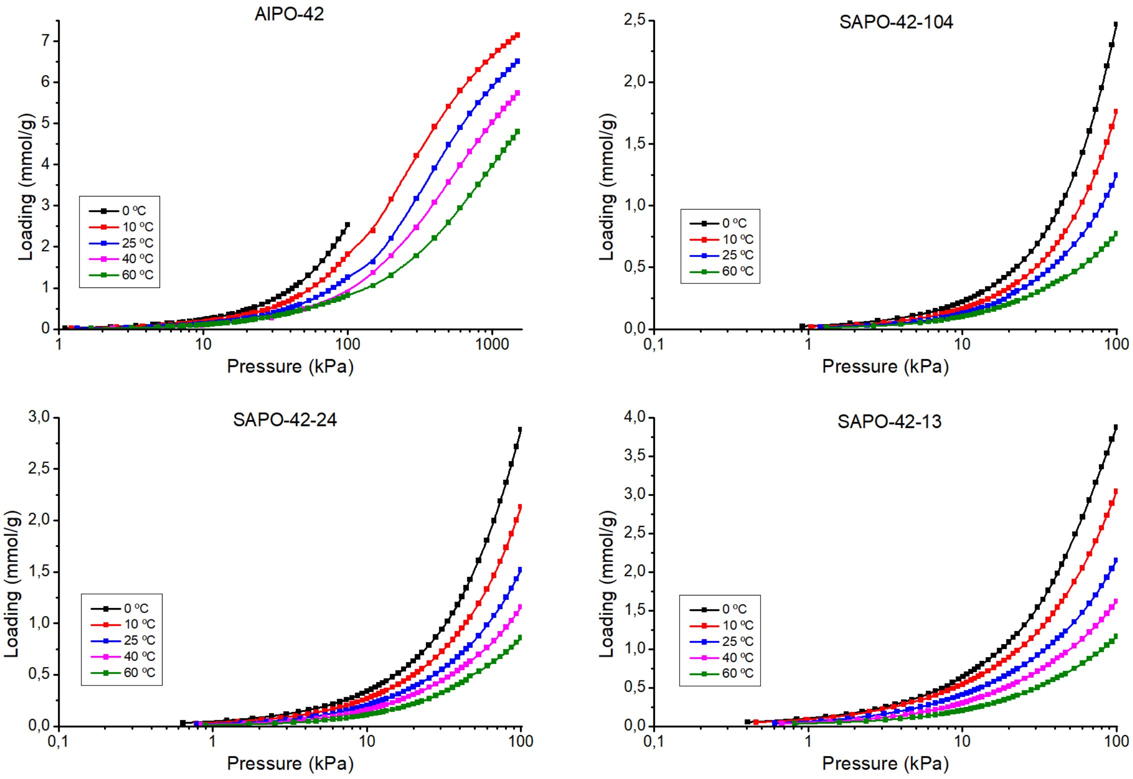

Figure S11. Adsorption isotherms of CO_2 on AlPO-42 and SAPO-42 materials.

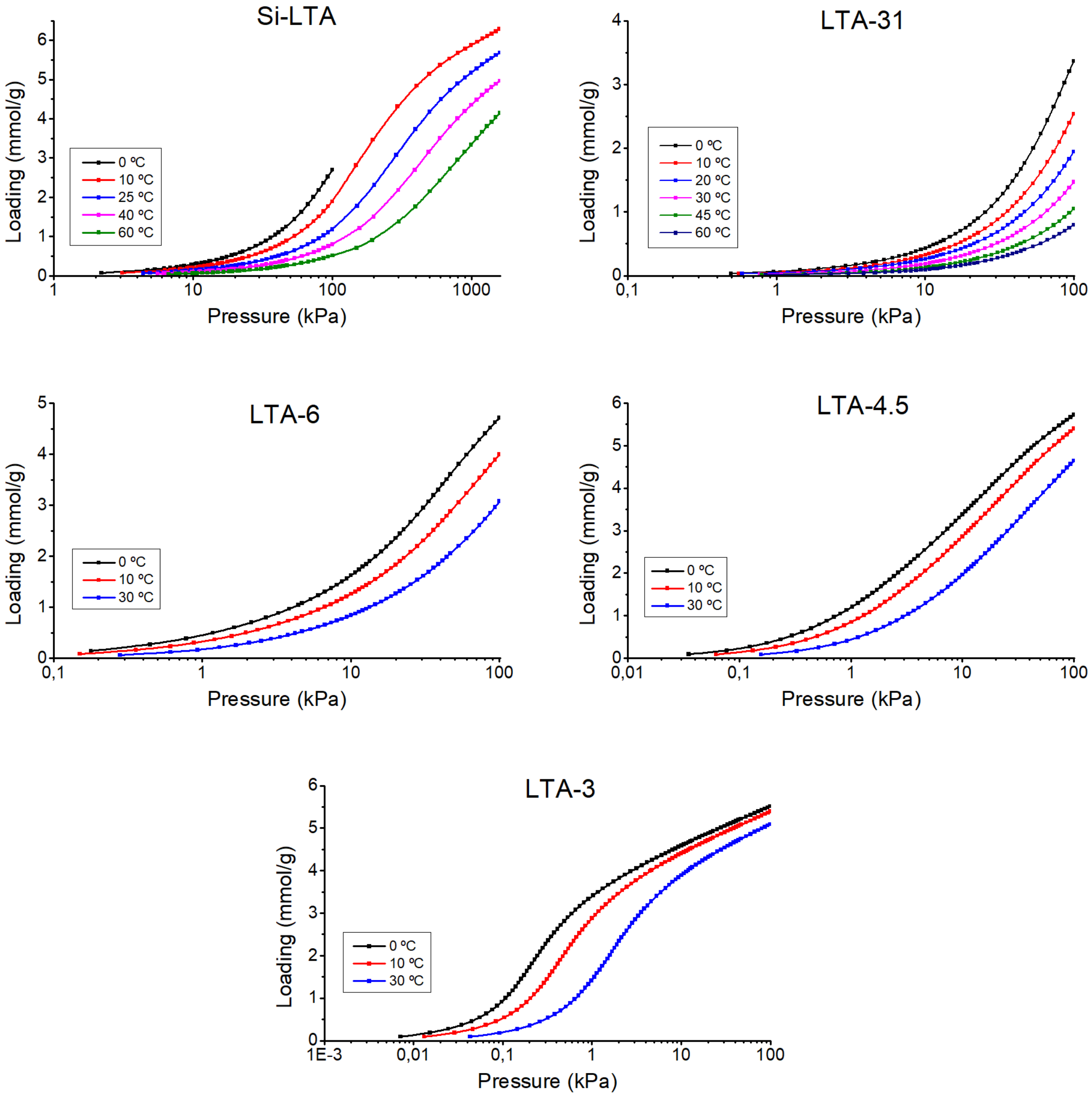

Figure S12. Adsorption isotherms of CO_2 on LTA zeolites.

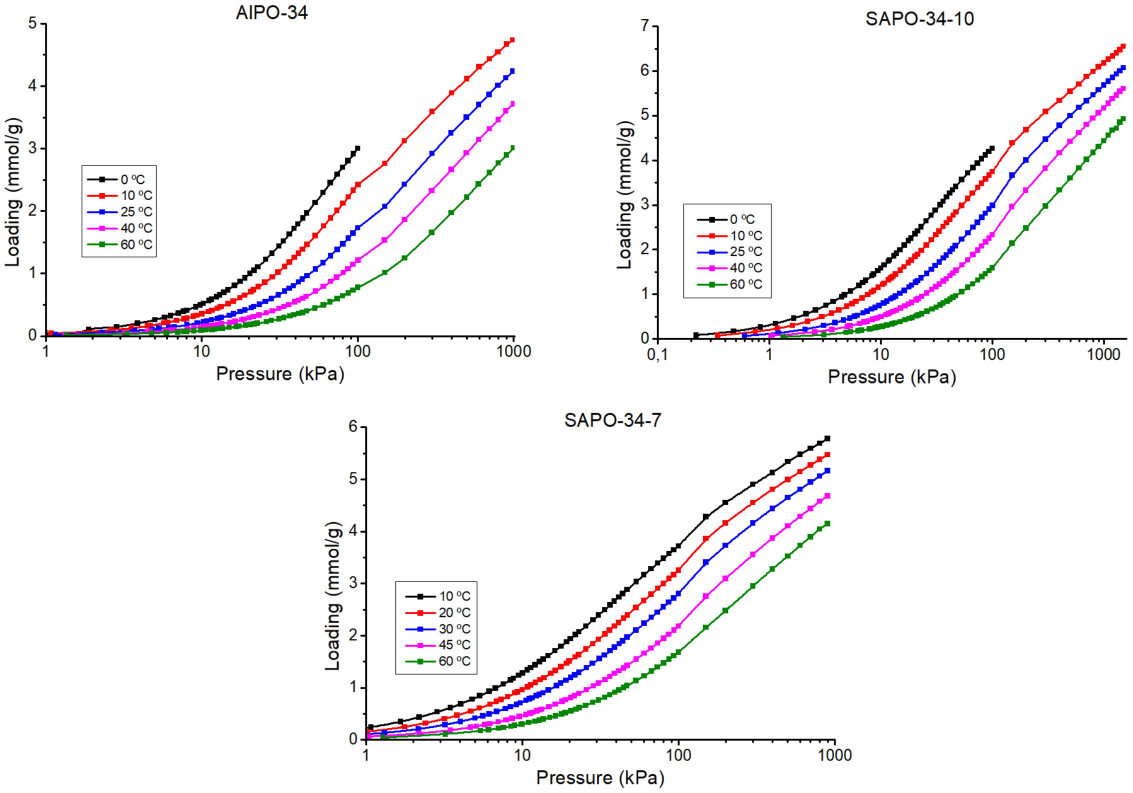

Figure S13. Adsorption isotherms of CO_2 on AlPO-34 and SAPO-34 materials.

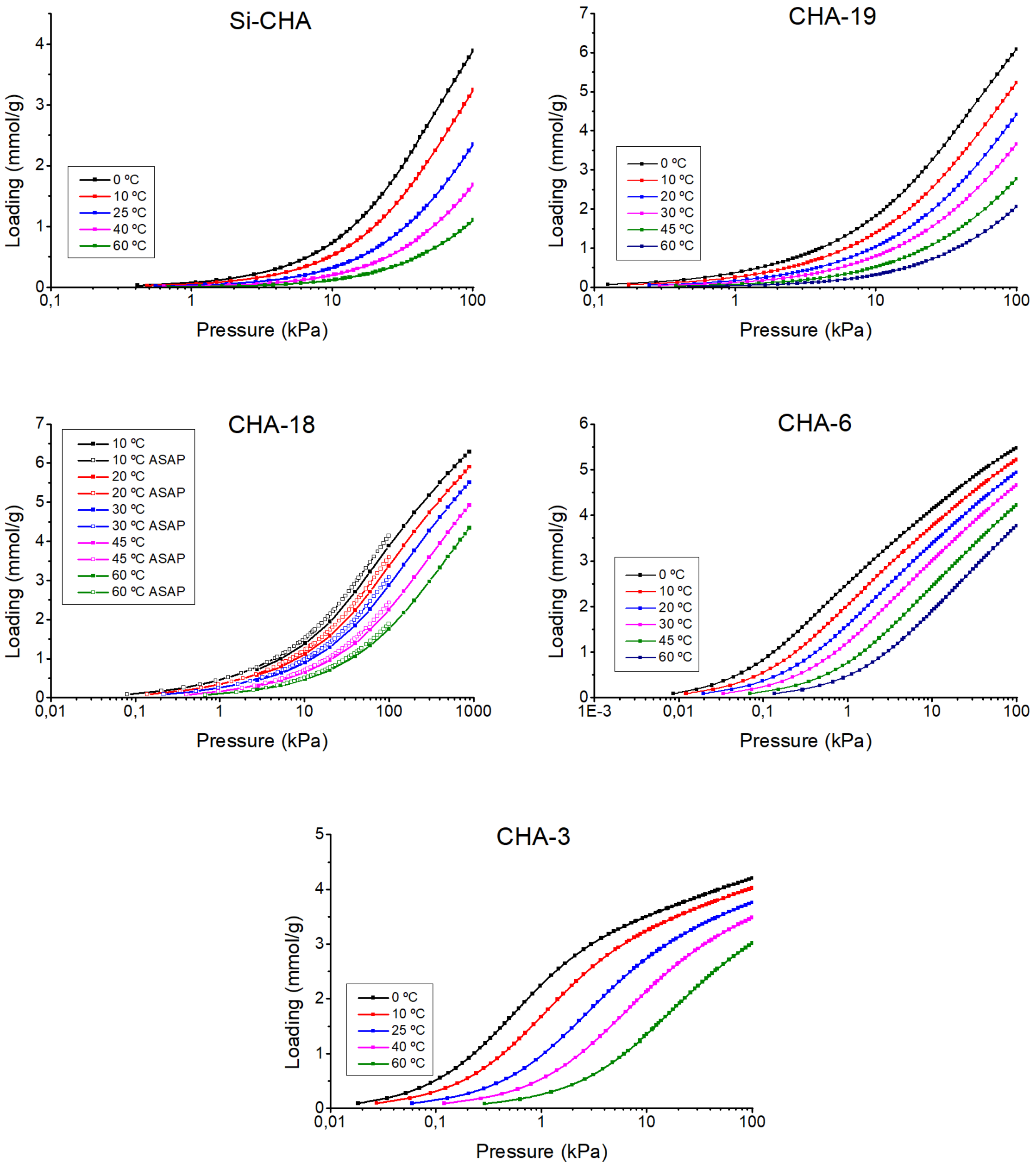

Figure S14. Adsorption isotherms of CO_2 on CHA zeolites.

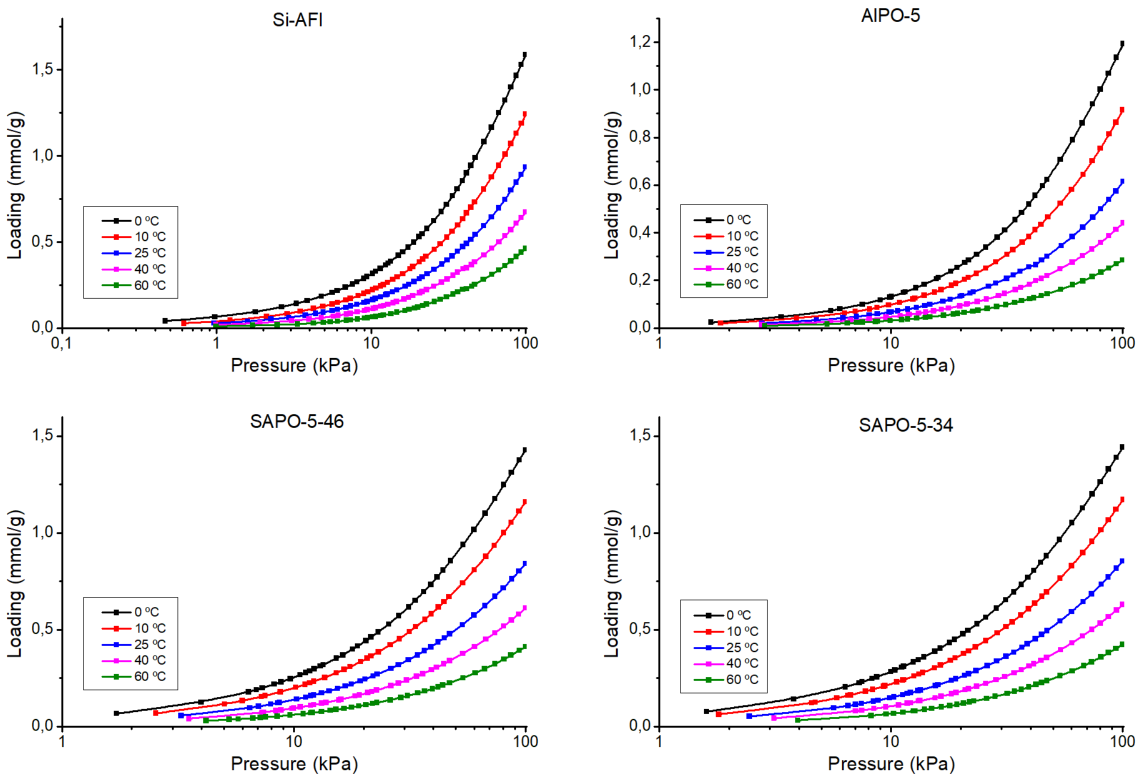

Figure S15. Adsorption isotherms of CO_2 on materials with AFI structure.

3.2 CH_4 Isotherms

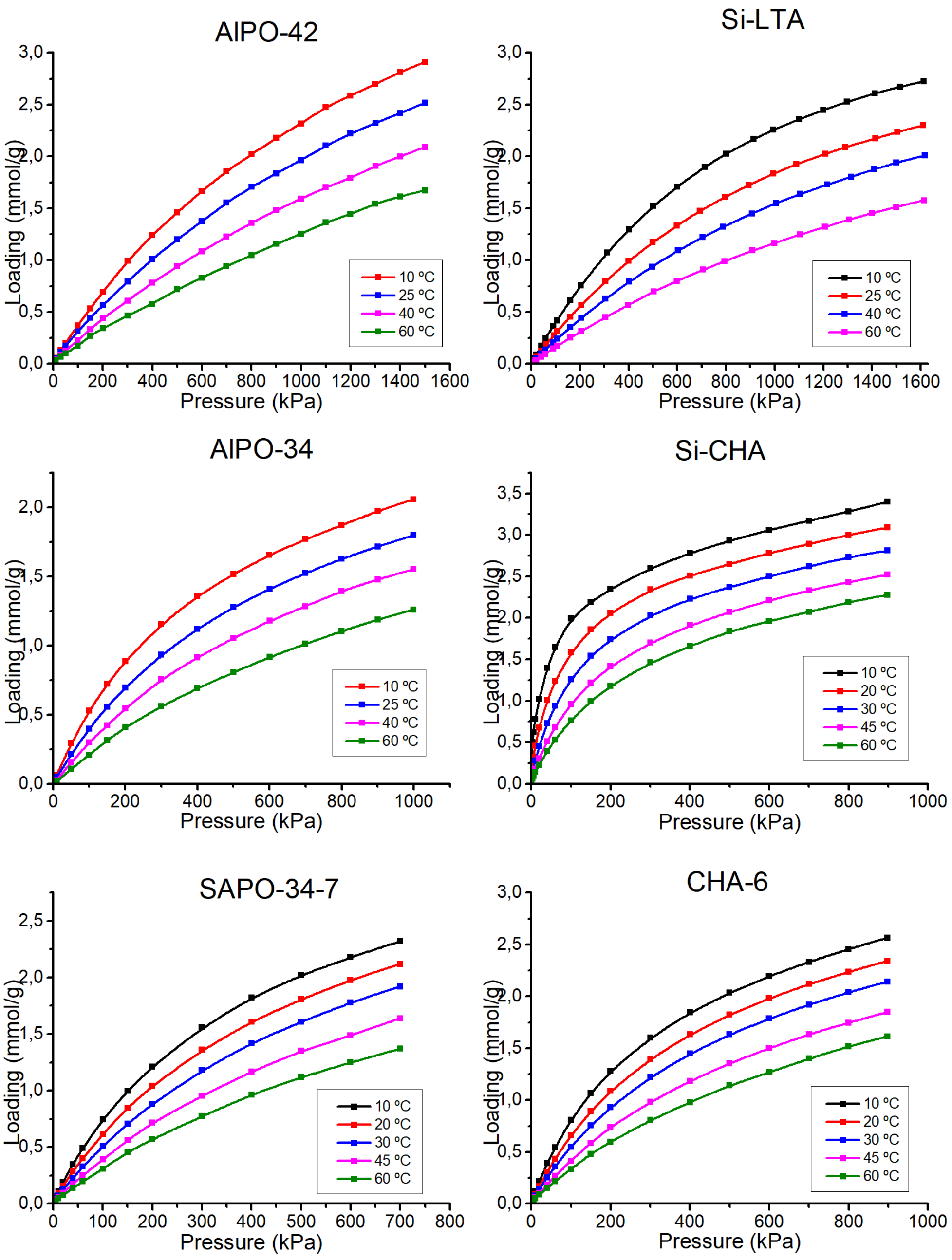

Figure S16. Adsorption isotherms of CH_4 on some materials.

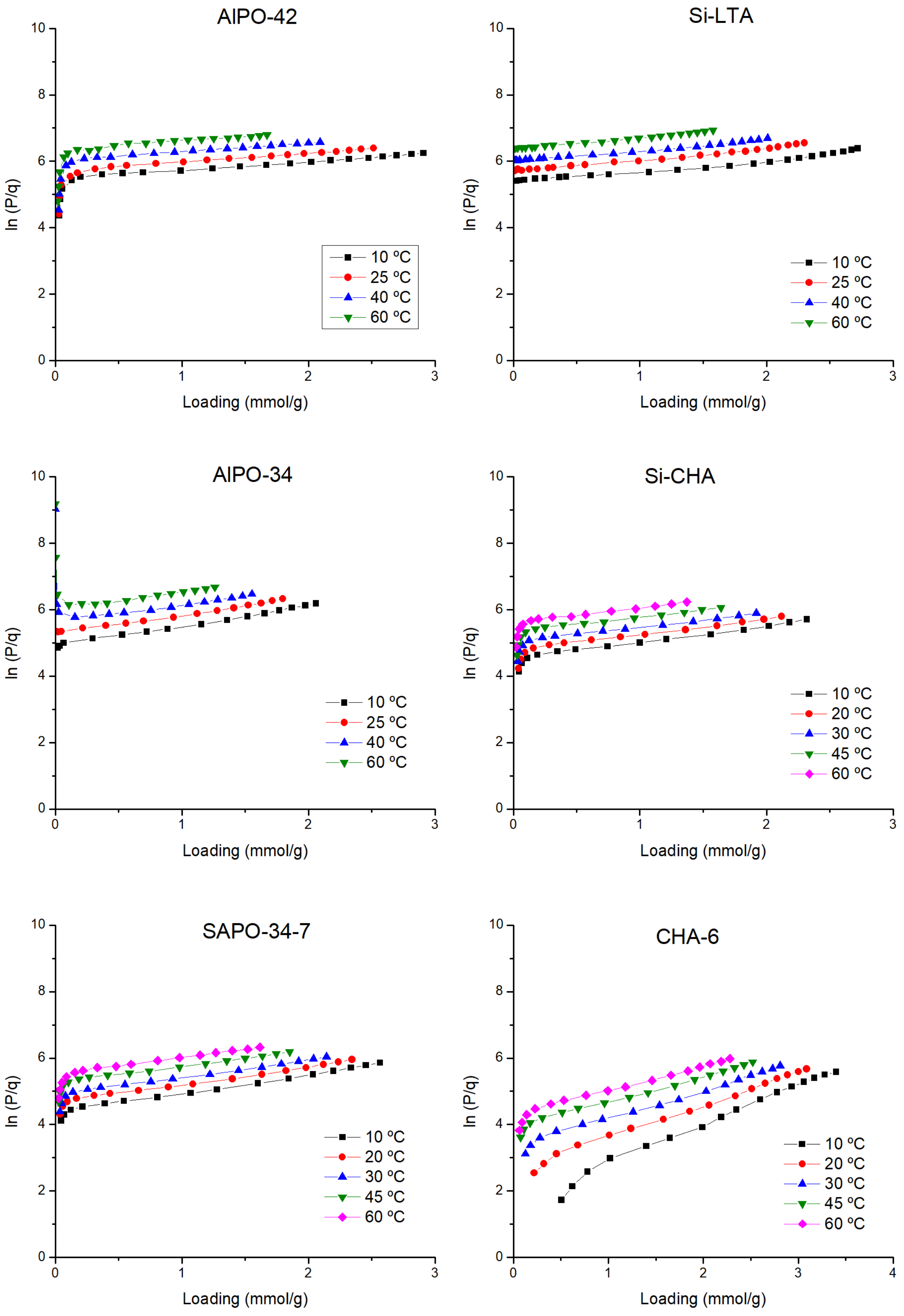

Figure S17. Virial plot of CH_4 isotherms of some materials.

4 REFERENCES

Bourgogne, M., Guth, J.-L., and Wey, R. (1985). Process for the preparation of synthetic zeolites, and zeolites obtained by said process. Available at: https://patents.google.com/patent/US4503024A/en.

Corma, A., Rey, F., Rius, J., Sabater, M. J., and Valencia, S. (2004). Supramolecular self-assembled molecules as organic directing agent for synthesis of zeolites. *Nature* 431, 287–290. doi:10.1038/nature02909.

Díaz-Cabañas, M.-J., and Barrett, P. A. (1998). Synthesis and structure of pure SiO2 chabazite: the SiO2 polymorph with the lowest framework density. *Chem. Commun.* 29, 1881–1882. doi:10.1039/a804800b.

Girnus, I., Jancke, K., Vetter, R., Richter-Mendau, J., and Caro, J. (1995). Large AlPO4-5 crystals by microwave heating. *Zeolites* 15, 33–39. doi:10.1016/0144-2449(94)00004-C.

IZA International Zeolite Association Synthesis Commission. Available at: http://www.iza-online.org/synthesis/default.htm.

Küehl, G. H. (1980). Preparation of shape selective zeolite alpha as catalyst.

Lemishko, T., Valencia, S., Rey, F., Jiménez-Ruiz, M., and Sastre, G. (2016). Inelastic Neutron Scattering Study on the Location of Brønsted Acid Sites in High Silica LTA Zeolite. *J. Phys. Chem. C* 120, 24904–24909. doi:10.1021/acs.jpcc.6b09012.

Martínez-Franco, R., Cantín, Á., Vidal-Moya, A., Moliner, M., and Corma, A. (2015). Self-assembled aromatic molecules as efficient organic structure directing agents to synthesize the silicoaluminophosphate SAPO-42 with isolated Si species. *Chem. Mater.* 27, 2981–2989. doi:10.1021/acs.chemmater.5b00337.

Martínez-Franco, R., Li, Z., Martínez-Triguero, J., Moliner, M., and Corma, A. (2016). Improving the catalytic performance of SAPO-18 for the methanol-to-olefins (MTO) reaction by controlling the Si distribution and crystal size. *Catal. Sci. Technol.* 6, 2796–2806. doi:10.1039/c5cy02298c.

Moscoso, J. G., Lewis, G. J., Gisselquist, J. L., Miller, M. A., and Rohde, L. M. (2003). Crystalline aluminosilicate zeolitic composition: UZM-9.

Nordstrand, R. A. Van, Santilli, D. S., and Zones, S. I. (1988). "An All-Silica Molecular Sieve That I s Isostructural with AlPO -5," in *Perspectives in Molecular Sieve Science*, eds. W. H. Flank and T. E. Whyte (Washington DC), 236–245. doi:10.1021/bk-1988-0368.

Palomino, M., Corma, A., Rey, F., and Valencia, S. (2010). New Insights on CO2−Methane Separation Using LTA Zeolites with Different Si/Al Ratios and a First Comparison with MOFs. *Langmuir* 26, 1910–1917. doi:10.1021/la9026656.

Prakash, A. M., and Unnikrishnan, S. (1994). Synthesis of SAPO-34: high silicon incorporation in

the presence of morpholine as template. *J. Chem. Soc. Faraday Trans.* 90, 2291. doi:10.1039/ft9949002291.

Schreyeck, L., Stumbe, J., Caullet, P., Mougenel, J.-C., and Marler, B. (1998). The diaza-polyoxa-macrocycle `Kryptofix222' as a new template for the synthesis of LTA-type AlPO4. *Microporous Mesoporous Mater.* 22, 87–106. doi:10.1016/S1387-1811(98)00082-1.

Young, D., and Davis, M. E. (1991). Studies on SAPO-5: synthesis with higher silicon contents. *Zeolites* 11, 277–281. doi:10.1016/S0144-2449(05)80232-5.

Zones, S. I. (1991). Conversion of faujasites to high-silica chabazite SSZ-13 in the presence of N,N,N-trimethyl-1-adamantammonium iodide. *J. Chem. Soc. Faraday Trans.* 87, 3709. doi:10.1039/ft9918703709.

Zones, S. I., and Van Nordstrand, R. A. (1988). Novel zeolite transformations: The template-mediated conversion of Cubic P zeolite to SSZ-13. *Zeolites* 8, 166–174. doi:10.1016/S0144-2449(88)80302-6.
